# Transcriptomic Analysis Reveal the Molecular Mechanisms of Wheat Higher-Temperature Seedling-Plant Resistance to *Puccinia striiformis* f. sp. *tritici*

**DOI:** 10.3389/fpls.2018.00240

**Published:** 2018-02-28

**Authors:** Fei Tao, Junjuan Wang, Zhongfeng Guo, Jingjing Hu, Xiangming Xu, Jiarong Yang, Xianming Chen, Xiaoping Hu

**Affiliations:** ^1^State Key Laboratory of Crop Stress Biology for Arid Areas, College of Plant Protection, Northwest A&F University, Yangling, China; ^2^Wuhan UnigueGene Bioinformatics Science and Technology Co., Ltd, Wuhan, China; ^3^NIAB East Malling Research (EMR), East Malling, United Kingdom; ^4^Agricultural Research Service, United States Department of Agriculture and Department of Plant Pathology, Washington State University, Pullman, WA, United States

**Keywords:** higher temperature, non-race-specific resistance, plant defense, plant-pathogen interaction, *Puccinia striiformis* f. sp. *tritici*, transcript profiling, wheat

## Abstract

Stripe rust, caused by *Puccinia striiformis* f. sp. *tritici* (*Pst*), is a destructive disease of wheat worldwide. The disease is preferably controlled by growing resistant cultivars. Wheat cultivar Xiaoyan 6 (XY 6) has been resistant to stripe rust since its release. In the previous studies, XY 6 was found to have higher-temperature seedling-plant (HTSP) resistance. However, the molecular mechanisms of HTSP resistance were not clear. To identify differentially expressed genes (DEGs) involved in HTSP resistance, we sequenced 30 cDNA libraries constructed from XY 6 seedlings exposed to several temperature treatments. Compared to the constant normal (15°C) and higher (20°C) temperature treatments, 1395 DEGs were identified in seedlings exposed to 20°C for 24 h (to activate HTSP resistance) and then kept at 15°C. These DEGs were located on all 21 chromosomes, with 29.2% on A, 41.1% on B and 29.7% on D genomes, by mapping to the Chinese Spring wheat genome. The 1395 DEGs were enriched in ribosome, plant-pathogen interaction and glycerolipid metabolism pathways, and some of them were identified as hub proteins (phosphatase 2C10), resistance protein homologs, WRKY transcription factors and protein kinases. The majority of these genes were up-regulated in HTSP resistance. Based on the differential expression, we found that phosphatase 2C10 and LRR receptor-like serine/threonine protein kinases are particularly interesting as they may be important for HTSP resistance through interacting with different resistance proteins, leading to a hypersensitive response.

## Introduction

Stripe (yellow) rust, caused by *Puccinia striiformis* f. sp. *tritici* (*Pst*), is a destructive disease affecting wheat production world wide (Wan, 2003; Chen, 2005). Breeding resistant cultivars is the best approach for controlling stripe rust (Zhang et al., 2001; Chen, 2007; Sui et al., 2009). Different types of stripe rust resistance have been identified and used for developing resistant cultivars (Chen, 2005, 2013). Based on specificity, resistance can be classified as race-specific and non-race-specific. Race-specific resistance is usually controlled by major genes and effective throughout plant development. However, new virulent *Pst* races can overcome race-specific resistance (Chen, 2005; Zheng et al., 2013). For example, the rapid development of *Pst* races that have overcome *Yr2, Yr9, Yr17*, and *Yr27* resistance has led to destructive epidemics in many parts of the world (Wellings, 2011). In contrast, non-race-specific resistance is usually quantitative and often controlled by several genes (Coram et al., 2008a; Chen, 2013).

High temperature resistance to *Pst* is activated by changes in temperature and is believed to be non-race-specific. Use of such temperature induced resistance could thus be considered as a durable method for managing stripe rust (Shang, 1998; Ma and Shang, 2000; Chen, 2013; Zhou et al., 2014). Resistance in both seedling and adult plants can be induced by temperature changes. High-temperature adult plant (HTAP) resistance has been successfully used to develop durable resistant cultivars in the United States since the early 1960s (Chen and Line, 1995; Line, 2002; Chen, 2005, 2013). Cultivars with only HTAP resistance are susceptible in seedlings when temperatures are low (diurnal temperatures changing from 4 to 20°C), but gradually become more resistant when plants grow older and temperatures are higher (diurnal temperatures changing from 10 to 30°C; Chen, 2013). HTAP resistance usually becomes visible after the tillering stage and reaches to the highest level on the flag leaves (Qayoum and Line, 1985; Milus and Line, 1986; Chen, 2013). Numerous genes or quantitative trait loci conferring HTAP resistance have been identified and used to develop wheat cultivars with durable resistance. HTAP resistance is generally partial and can have a wide range of levels, depending on individual genes and the number of genes in a cultivar (Chen, 2013). Although HTAP resistance is influenced by temperature and growth stage, different HTAP resistance genes may have different sensitivities to temperature and/or plant growth stage. Similar to HTAP resistance, higher-temperature seedling-plant (HTSP) is also induced by higher-temperature. However, typical HTSP resistance is not affected much by plant growth stages. HTAP resistance is often reversible as plants become susceptible or less resistant when temperature changes from high to low (Qayoum and Line, 1985; Chen, 2013). In contrast, at least with the wheat cultivars studied, seedlings with HTSP resistance continue showing resistance after exposure to 18~21°C for only 24 h (Lu and Li, 1958; Lu, 1996; Ma and Shang, 2000; Hu X. P. et al., 2012; An et al., 2015).

Winter wheat cultivar Xiaoyan 6 (XY 6), developed from a cross between a wheat (*Triticum aestivum*) cultivar and *Elytrigia elongatum* (Li, 1986), has shown partial resistance to stripe rust, and the resistance has been characterized as HTSP resistance (Ma and Shang, 2000; Hu X. P. et al., 2012). An et al. (2015) found that the treatments of 18–24°C after inoculation of seedlings significantly reduced infection type and uredospore production compared to the seedlings grown at constant 16°C. At 8 days after inoculation when plants had the mosaic symptom without sporulation, the plants exposed to the optimal temperature of 20°C for 24 h showed incompatible reaction, in contrast to the compatible reaction on the plants without the higher temperature treatment. These results show that XY 6 has HTSP resistance to stripe rust and this type of resistance is induced by higher-temperatures.

HTAP resistance has been found to involve different mechanisms. The HTAP resistance controlled by *Yr36* can be observed at the seedling stage under high temperatures, but the highest level of resistance is expressed at the adult-plant stage at high temperatures (Uauy et al., 2005; Chen, 2013). Originally from *Triticum dicoccoides, Yr36* encodes a predicted kinase and a steroidogenic acute regulatory protein-related lipid transfer (START) domain (Fu et al., 2009). Both the kinase and START domains are necessary for the resistance function. Temperature and *Pst* inoculation consistently up-regulates expression of the resistance alleles, but down-regulates the susceptible alleles. The START domain has the ability to bind lipids from stripe rust fungus at high temperature and change its conformation, which may cause the kinase domain to initiate a signaling cascade leading to programmed cell death. *Yr18* (also known as *Lr34*) is considered as a HTAP resistance gene as the level of resistance is increased by high temperatures (Chen, 2013). This gene encodes a putative ATP-binding cassette (ABC) transporter (Krattinger et al., 2009a,b). This drug resistance gene product contains two cytosolic nucleotide binding domains (NBD) and two hydrophobic transmembrane domains. The pleotropic resistance gene in wheat confers non-race-specific resistance to stripe rust, leaf rust, stem rust and powdery mildew, and also confers resistance to other diseases when transferred into barley, corn, rice and other plant species (Krattinger et al., 2011, 2013, 2016; Risk et al., 2013). *Yr46* is also a pleiotropic gene, providing adult-plant resistance to stripe rust, leaf rust (*Lr67*), stem rust (*Sr55*) and powdery mildew (*Pm46*) (Herrera-Foessel et al., 2011; Chhetri et al., 2016). This gene encodes a hexose transporter that differs from the susceptible form of the same protein by just two conserved amino acids (Moore et al., 2015). The susceptible allele functions as a higher affinity glucose transporter, while the resistant allele has a dominant-negative effect through heterodimerization with functional transporters to reduce glucose uptake. These cloned adult-plant or HTAP resistance genes do not have LRR domains and do not show race specificity. However, nine NBS-LRR genes are involved in *Yr39*-controlled HTAP resistance based on transcriptomics analyses (Lin and Chen, 2007; Coram et al., 2008a). In addition to HTAP resistance, transcriptomics analyses have been used to study mechanisms of race-specific all-stage resistance and numerous genes with diverse functions have been found to be involved in this type of resistance (Coram et al., 2008b, 2010; Chen et al., 2013; Zhang et al., 2014; Hao et al., 2016). The effects of temperature on plant defense have been studied for other diseases (Wang et al., 2017). In contrast, there were no reports on molecular mechanisms on HTSP resistance to stripe rust, and the identity of genes and biochemical pathways involved in HTSP resistance were unknown before the present study.

The main objective of this study was to identify co-regulated genes that show significant changes in expression patterns related to HTSP resistance. We confirmed that exposure of XY 6 seedlings to 20°C for 24 h was sufficient for activating the resistance to *Pst*, and used this exposure regime to study gene expression during the activation of HTSP resistance in comparison with the inoculated seedlings grown at constant temperatures of 15 and 20°C. Through the comparison, we identified a large number of differentially expressed genes (DEGs) induced by the higher-temperature treatment. These DEGs allowed us to infer the mechanisms underlying HTSP resistance.

## Materials and methods

### Plant materials, growth conditions, and temperature treatments

Chinese yellow rust race 32 (CYR32) was used to inoculate wheat cv. XY 6 (susceptible but possessing HTSP) and Mingxian 169 (MX 169, susceptible without HTSP). Seeds (10–15) were sown in plastic pots (10 × 10 × 10 cm^3^) at a seed-to-seed distance of ca. 1.5 cm. Urediniospores of CYR32 were added to sterile water at a ratio of ~1:6–9 (v/v) and stirred with a vaccination needle; urediniospores were floating at the top of water surface and this spore suspension was then used to inoculate seedlings. For each cultivar, a total of 120 pots (90 inoculated and 30 not inoculated) were used for each of three biological replicate experiments over time. At the one-leaf stage (~10 days after sowing), seedlings were inoculated with the urediniospore suspension using a paint brush and then kept in a growth chamber (Percival E-30B, Perry, IA, USA) in dark at 10 ± 1°C and 80% relative humidity for 24 h, as described previously (Wang et al., 2017). Thereafter, the inoculated seedlings were divided into three groups for exposure to different temperature regimes. The first group was maintained at 15 ± 1°C [the normal temperature treatment (N)]; the second group was for the normal-higher-normal temperature treatment (NHN) – seedlings were kept at 15 ± 1°C from two to eight days after inoculation (dpi) and then transferred at 20 ± 1°C for 24 h, and finally moved back to 15 ± 1°C. This regime was shown previously to activate HTSP in XY 6 (An et al., 2015). The third group was maintained at 20 ± 1°C (H) 24 h after *Pst* inoculation. Wheat plants inoculated with sterile water were used as controls (Figure S1). The first sampling time (0 h) corresponded to the beginning of NHN treatment (at 8 dpi, i.e., 192 h after *Pst* inoculation). Leaf tissue from seedlings under three temperature treatments was sampled at 0, 12, 24, 48, and 120 h after temperature treatment was imposed (i.e., 192~312 h after *Pst* inoculation). For each cultivar, leaf tissue samples were collected at each time point for each biological replicate under each treatment.

### Histopathological analysis

*Pst* inoculated leaves (XY 6 and MX 169) sampled at 12, 24, 48, and 120 h were assessed for the number of necrotic cells per infection site, the length of uredinium and the number of uredinia per leaf under a microscope. The number of necrotic cells per infection site and the length of uredinium were measured based on a published method (Wang et al., 2017). To measure the number of uredinia per leaf, 10 leaves were randomly selected for each of the three biological replicates. Microscopic observations were performed using an Olympus BX-51 microscope (Olympus Corporation, Tokyo, Japan) or an Olympus SZ-PT anatomical lens (Olympus Corporation, Tokyo, Japan); the data were measured using Cell Sens Entry software (v.1.6). The length and number of uredinia per leaf as well as the number of necrotic cells were analyzed using analysis of variance (ANOVA) and multiple comparison tests, which were performed using a generalized linear model with a Poisson distribution and the Tukey test by the *glm* and *glht* functions of R software (v.3.2.3), respectively.

### Establishment and sequencing quality evaluation of cDNA libraries

The leaves of XY 6 sampled at 0 and 24 h (8 and 9 dpi, respectively) were used for RNA-Seq (Figure S1). Samples at 0 h from N and NHN treatments were the same, as the samples had not yet been subjected to higher temperature. For each of the three repetitions, one sample was selected and RNA was extracted under each combination of treatment and time point, a total of 30 samples from XY 6 were collected to extract RNA. RNA extracting was conducted using the PureLink® Plant RNA Reagent (Invitrogen, Carlsbad, CA, USA) and then treated with DNase I (Thermo Fisher, Waltham, MA, USA) at a concentration of 1 U/mg. The quality and concentration of extracted RNA were checked using an Agilent 2100 Bioanalyzer (Agilent Technologies, Waldbronn, Germany). Thirty paired-end (PE) cDNA libraries were sequenced separately and PE reads were generated separately with Q30 as a base phred quality score threshold (Each library > 4 Gb and single plexing). Sequencing was performed on each library from each sample to generate 100 bp PE reads for transcriptome sequencing on an Illumina High-Seq 2000 platform. Library construction was accomplished using commercial products (Illumina); sequencing was done by Macrogen (Seoul, South Korea).

### Optimization and evaluation of CDMC assembly strategy

RNA-Seq data trimming and adapter clipping were performed using Trimmomatic (v.0.33; Bolger et al., 2014). Reads were assembled by *de novo*, combined with reference-based mapping using CD-HIT-EST (CDMC) to remove redundancy (Li et al., 2013). The *de novo* process was conducted by reconstructing a transcript library with reads using Trinity (v.2.0.6; Haas et al., 2013). The mapping process was carried out by mapping the reads to the Chinese Spring wheat genome (*T. aestivum* ftp://ftp.ensemblgenomes.org/pub/release-8/plants/fasta/triticum_aestivum/dna/) using Tophat (v.2.1.0). Then, a transcript library was reconstructed using Cufflinks (v.2.2.1) and Coffmerge (v.2.2.1; Trapnell et al., 2012). The CDMC process was conducted by combining the above two libraries constructed from *de novo* and mapping strategies using CD-HIT-EST (v.4.6.4) with a similarity threshold of 95% identity (Fu et al., 2012) to generate a new transcript library.

### Analysis of differentially expressed genes

The normalization factors were calculated using the trimmed mean of *M*-values (TMM) method of RSEM (v.1.1.17; Li and Dewey, 2011). The TMM-FPKM value was the expression level of each transcript expressed as the fragments per transcript kilobase per million fragments mapped value by the TMM normalization of the RNA-Seq data. In this study, we focused on DEGs during the induction process of HTSP resistance to *Pst*. Thus, significant DEGs over time were not analyzed. To eliminate the time effect, the 0 h samples were used as background. TMM-FPKM values for 24-h samples were divided by the corresponding values for 0 h samples, to avoid division by zero, 1 was added to each value (Oono et al., 2013). To validate whether data met the requirement for a normal distribution, the Kolmogorov-Smirnov test was used (SPSS software, v.20; Table S1). HTSP resistance to *Pst* was induced by two factors (higher temperature and inoculation with *Pst*); thus the interaction between these two factors should be considered. Therefore, the DEGs were evaluated using the following model:

(1) $$\begin{array}{r} y_{i}=\mu +\alpha B+\beta_{I}I+\beta_{T}T+\beta_{IT}IT+\varepsilon_{i} \end{array}$$

where *y*_*i*_ is the expression value for gene *i*; μ is the population mean expression; *B, I* and *T* are the indicator variables that describe the batch, inoculation and temperature treatment, respectively; α, β_*I*_, and β_*T*_ are the batch effect, inoculation effect and temperature effect, respectively; IT is the interaction term for inoculation and temperature; β_*IT*_ is the IT interaction effect; and ε_i_ is the random error following a normal distribution with mean of 0 and variance of σ^2^. The model terms were tested by ANOVA, and *P*-values for all genes were adjusted using a false discovery rate (FDR) of α < 0.05 (Benjamini and Hochberg, 1995). In addition, a log_2_-fold change > 1 or < −1 and a logCPM (log_2_ counts per million) > −2 were used as additional criteria to select DEGs (McCarthy et al., 2012; Liu Y. et al., 2014).

### Identification of chromosomes of DEGs

Identified DEGs were mapped to the hexaploid wheat genome of cv. Chinese Spring (CS) (ftp://ftp.ensemblgenomes.org/pub/release-8/plants/fasta/triticum_aestivum/dna/). These mapped DEGs were categorized into two groups: (1) DEGs with chromosome location information from the reference wheat genome of CS and (2) DEGs without location information mapped from the *de novo* assembly were used as probes for use with BLASTN (with an *E*-value < 1E−50) against the predicted mRNA database of the CS genome to search location information on chromosomes (Laudencia-Chingcuanco et al., 2006). A circles-plot was made using R software (v.3.2.3).

### Protein-protein interaction (PPI) network analysis

Proteins encoded by identified DEGs were used to analyse protein-protein interactions (PPIs) based on the STRING database of the model plant *Arabidopsis thaliana* (with an *E-*value < 1E−10) (http://string-db.org/). PPIs with combined confidence scores greater than 0.7 were selected (Franceschini et al., 2013; Liu et al., 2016) and visualized with CYTOSCAPE (v.2.8, http://cytoscape.org/; Shannon et al., 2003).

### Functional annotation and enrichment analysis

For functional annotation, transcripts were subjected to BLASTX (v.2.2.28, *E-*value < 1E−5) analysis against several protein databases, including the Nr, Swiss-Prot and KEGG databases. The transcripts were named according to the annotation in the Nr database. BLAST2GO (Conesa et al., 2005) was then used to obtain GO annotations. To investigate the metabolic pathways of those annotated transcripts, the transcripts were aligned to the KEGG database. PPI network, GO terms and KEGG pathways with FDR(BH adjustment) corrected *P-*values smaller than 0.05 were considered statistically significant. *R* genes in DEGs were predicted (*E-*value < 1E−5) based on the information from the Plant Resistance Gene Database (Sanseverino et al., 2013). Transcription factors (TFs) in DEGs were predicted (*E-*value < 1E−5) according to the Plant Transcription Factor Database (Jin et al., 2014).

### Quantitative reverse-transcription-PCR analysis

Twelve transcripts were randomly selected for qRT-PCR analysis (Table S2). UltraSYBR Mixture (Kangwei, Beijing, China) and iQ™ 5 (Bio-Rad, Hercules, CA, USA) were used for qRT-PCR analysis of all reactions according to the manufacturer's instructions. Data were collected from three replicate experiments—the samples used for qRT-PCR were the same as those used for RNA-Seq, each consisting of at least three technical repeats. Negative controls that lacked templates were also included. The amplification efficiency of primers was determined (Figure S2) using LinReg PCR (Ramakers et al., 2003). The wheat ATP-dependent 26S proteasome regulatory subunit (26S) and cell division control (CDC) genes were chosen as internal reference genes for each qRT-PCR assay (Paolacci et al., 2009; Scholtz and Visser, 2013). The relative expression of selected transcripts was calculated using the 2^−ΔΔCT^ method (Livak and Schmittgen, 2001).

### RNA-seq data submission

The raw data used in the present study for transcriptome assembly and gene expression analysis have been submitted to the NCBI Sequence Read Archive (SRA) database under accession numbers from SRR5580869 to SRR5580898.

## Results

### Histopathological observation of *Pst* infections

**Figure 1:**
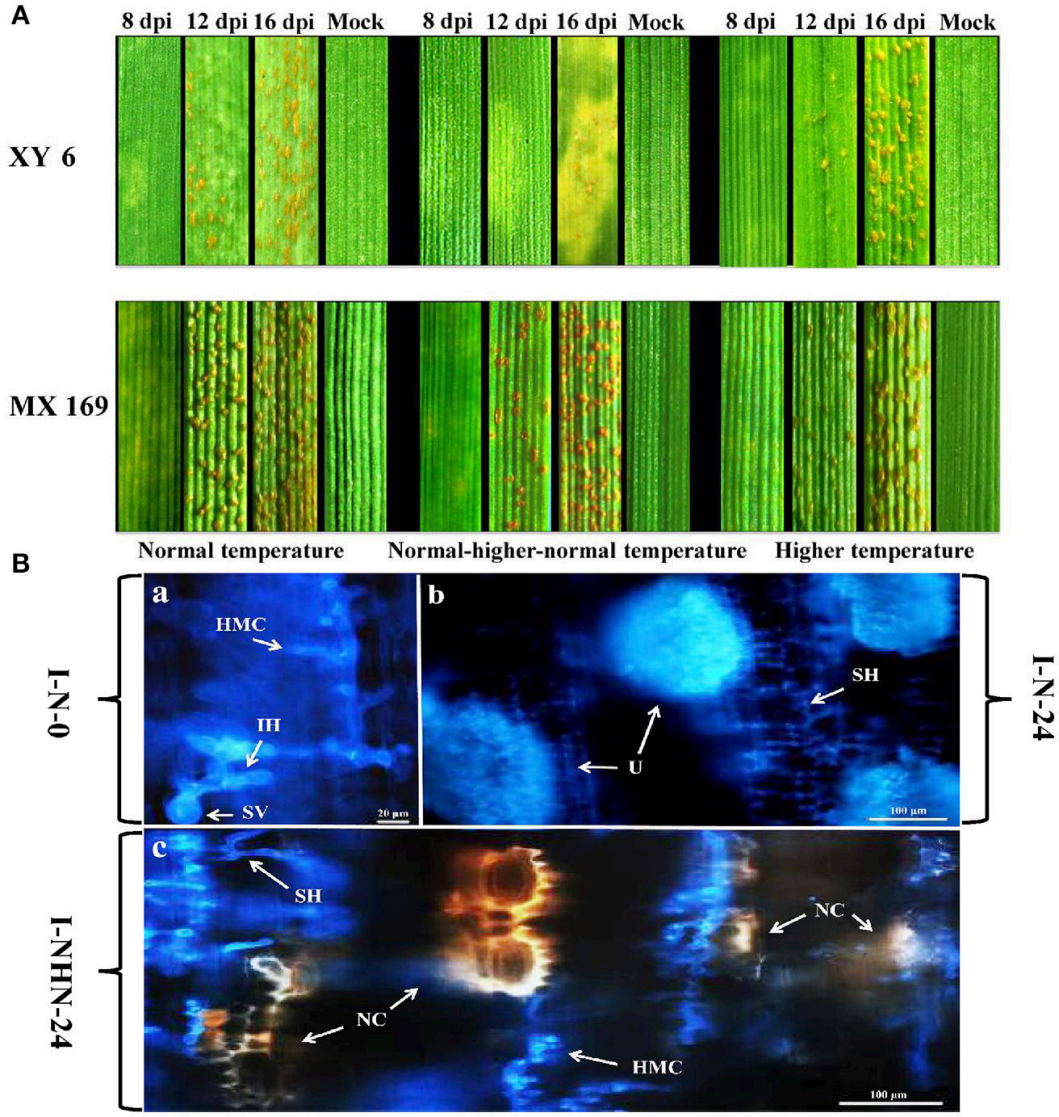
Histopathology observation during *Puccinia striiformis* f. sp. *tritici* infection in XY 6 (susceptible but possessing HTSP) and MX 169 (susceptible without HTSP) under different temperature treatments. **(A)** The infection types on XY 6 and MX 169 under different temperature treatments were observed at 8, 12 and 16 days post-inoculation (dpi). **(B)** Leaves of XY 6 infected by *Puccinia striiformis* f. sp. *tritici* at normal temperature 0, 24 h (I-N-0 and I-N-24), normal-higher-normal temperature 24 h (I-NHN-24), are examined under an epifluorescence microscope. I-N-0: The substomatal vesicle (SV), infection hypha (IH), secondary hyphae (SH), and haustorial mother cells (HMC) formed at the infection site at 0 h under the N treatment. I-N-24: SH formed at the infection site, which extends rapidly and formed larger colonies, and then further produce a large number of uredinia (U) at 24 h under the N treatment. I-NHN-24: SH formed at the infection site at 24 h and further induces the necrosis of host cell (NC) under the NHN treatment. 0~24 h means the hours post-temperature treatment which represents 192~216 h after inoculation. Bars, 20~100 μm. The samples of I-N-0 and I-NHN-0 are same before the temperature treatment. The results of H treatment have not been shown, because the results are similar to the N treatment.

**Figure 2:**
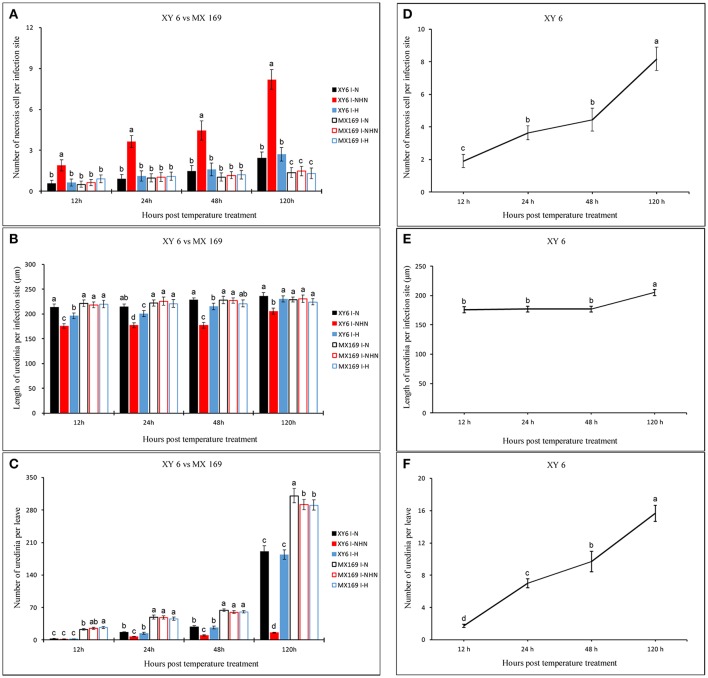
The influence of *Puccinia striiformis* f. sp. *tritici* development and the wheat response among different temperature treatments (black, red and blue represent normal temperature (N), normal-higher-normal temperature (NHN) and higher temperature (H) treatment, respectively) during the initial symptom expression stage of stripe rust development in XY 6 (Filled histograms) and MX 169 (Open histograms). 12~120 h means the hours post-temperature treatment which represents 204~312 h after inoculation. **(A)** The number of necrosis of host cell, **(B)** Linear length of uredinia and **(C)**. The number of uredinia per leaf. The lowercase letters means significant difference between different temperatures treatment in the different cultivars at the same time. The **(D–F)** are necrosis number of host cell, linear length of uredinia and the number of uredinia per leaf at different time points under NHN treatment in XY 6, respectively. For each treatment, the error bars are the mean ± SE of three replicates.

Among the normal temperature (N), normal-higher-normal temperature (NHN), and higher temperature (H) treatments, the necrosis on leaves at 16 dpi was observed only in cv. XY6 from the NHN treatment. There was no change in the infection type in cv. MX 169 in all three treatments (Figure 1A). After exposure to 20°C for 24 h, necrosis of host cells (NC) was observed around secondary hyphae (SH) at the infection site in the NHN treatment of XY 6 (Figure 1B). XY 6 had more (*P* < 0.05) necrotic cells per infection site, shorter (*P* < 0.05) uredinial length, and fewer (*P* < 0.05) uredinia per leaf than MX 169 in the NHN treatment at all-time points (Figures 2A–C,E). In addition, the number of necrotic cells per infection site and number of uredinia per leaf in XY 6 were greater (*P* < 0.05) in the NHN 24 h treatment than in the NHN 12 h (Figures 2D,F). These results confirmed that HTSP resistance was activated and that the hypersensitive response (HR) of XY 6 to *Pst* was induced by exposure to 20°C for 24 h.

### Identification of DEGs and their chromosome locations

A total of 65.95 million 101-bp paired-end clean reads were obtained, 91.23% of which had quality scores at or greater than the Q30 level (Table S3). The CDMC assembly strategy yielded 445226 transcripts with an N50 length of 1,849 bp (Table S4). Approximately 81.1 and 2.8% of the transcripts were longer than 400 bp and 4,000 bp, respectively (Figure S3A). About 37.0 and 3.4% of the transcripts with a predicted open reading frame (ORF) were longer than 300 and 1,000 bp, respectively (Figure S3B).

**Figure 3:**
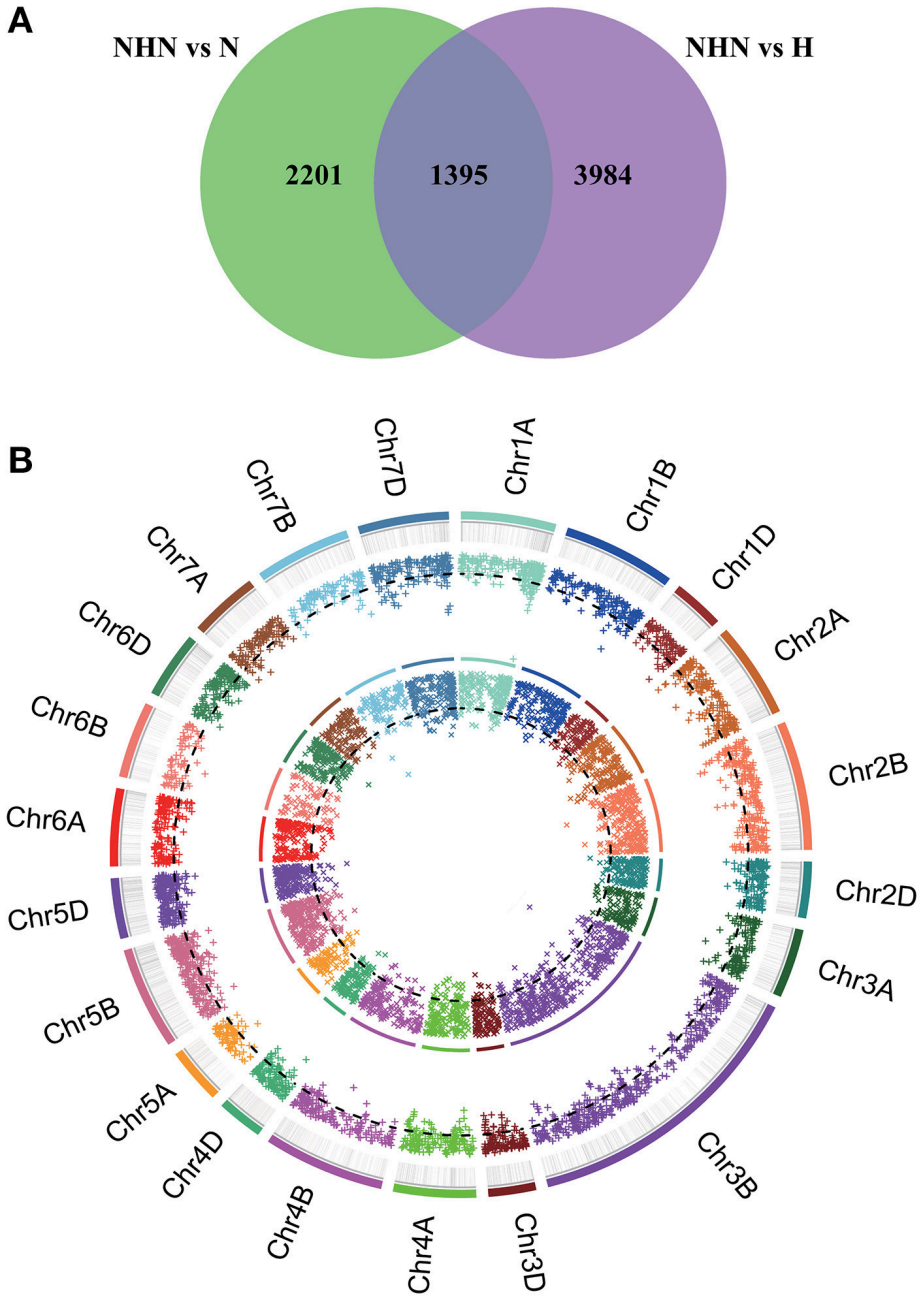
The distribution features of differentially expressed genes (DEGs) under different temperature treatment models. **(A)** The distribution features of DEGs (I^*^T) under normal (N), normal-higher-normal (NHN) and higher (H) temperature treatments. **(B)** Circles-plot of DEGs location in Chinese spring wheat genome. The circles from outside going in represent chromosomes (different colors represent different chromosomes) and each black short line at outside circle represents one DEGs gene. The first outside imaginary line circle is the cluster of false discover rate (FDR) values of DEGs for NHN vs. H (I^*^T) treatment, the second is the cluster of FDR values of DEGs for NHN vs. N (I^*^T) treatment. The FDR values of DEGs in inner circles are higher than those in the outer circles. The black dotted line represents threshold (FDR = 0.01).

To identify genes with differential expression during the process of HTSP resistance induction, a linear model (Equation 1) was used to analyse the RNA-Seq data assembled using the CDMC strategy. Significant differences between NHN and N treatments, and between NHN and H treatments were found for 3596 (1788 up-regulated, 1808 down-regulated) and 5379 (2278 up-regulated, 3101 down-regulated), respectively (Table S5). Functions for ca. 20% of the DEGs were unknown based on the Nr database. Among the 8975 DEGs, 1395 were identified in both NHN-N and NHN-H comparisons. Thus, there were 7580 unique DEGs, of which 2201 and 3984 expressed in the N and H treatments, respectively (Figure 3A).

Chromosome location information was obtained for 3731 of the 7580 unique DEGs. They were located on B chromosomes (41.1%), D chromosomes (29.7%) and A chromosomes (27.2%). Among the B chromosomes, 12.3, 8.7, 8.0, 4.4%, 4.3, 3.0, and 1.9% of the DEGs were on 3B, 5B, 2B, 4B, 1B, 7B, and 6B, respectively (Data S1; Figure 3B).

### Verification of RNA-seq analysis by qRT-PCR

**Figure 4:**
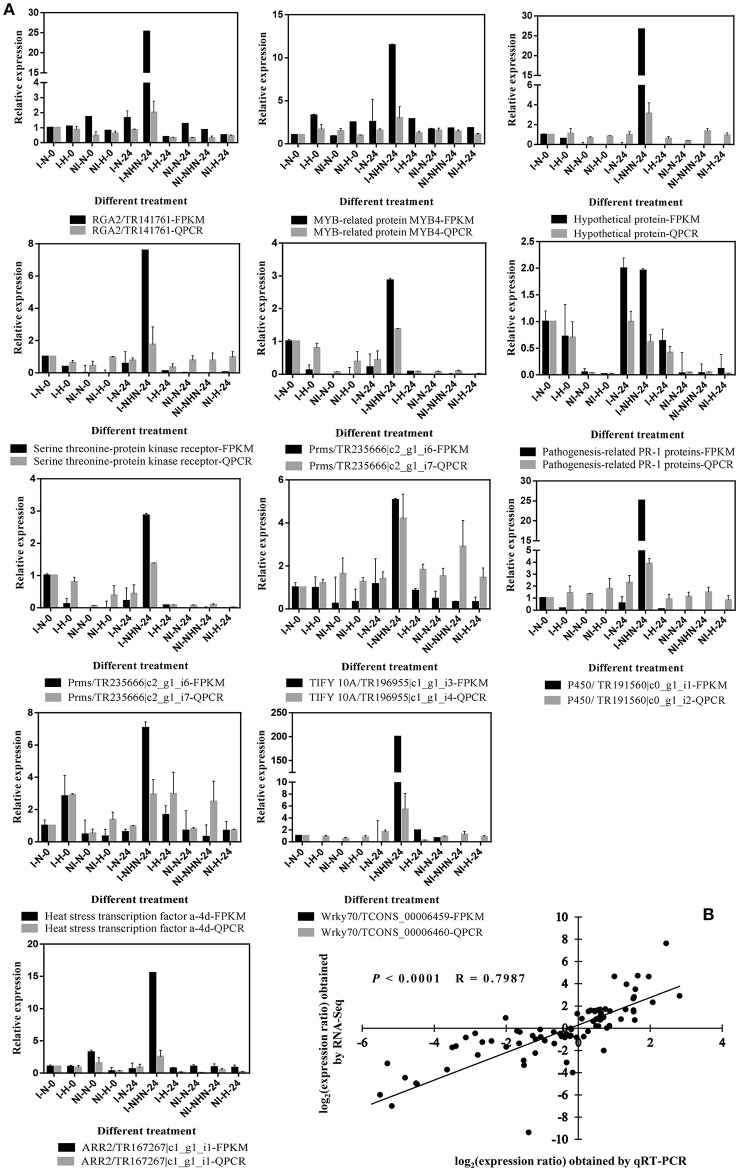
Verification of RNA-Seq analysis by qRT-PCR. **(A)** Relative expression levels of 12 randomly selected transcripts are verified through qRT-PCR. The gray histograms represent the relative gene expression levels analyzed using qRT-PCR. The black histograms represent TMM-FPKM values which are the relative expression levels of RNA-Seq data. The error bars are the mean ± SE of three replications. **(B)** Comparison between the log_2_ of expression ratios of DGEs obtained from RNA-Seq and qRT-PCR.

To verify the gene expression profiles from the RNA-Seq analysis, 12 transcripts were randomly selected for qRT-PCR analysis. The I-N-0 sample was used as a control when calculating relative expression levels. RNA-Seq (TMM-FPKM) and qRT-PCR results are shown in Figure 4. The relative expression levels of the transcripts from qRT-PCR were nearly identical to those from the RNA-Seq data (Figure 4A), the correlation coefficient was 0.80 (*P* < 0.0001; Figure 4B).

### Functional annotation and enrichment analysis of DEGs

For the NHN treatment, 1395 DEGs were enriched in KEGG pathways. Notably, DEGs in ribosome metabolism (KO 03010), plant-pathogen interaction (KO 04626) and glycerolipid metabolism (KO 00561) were significantly enriched (*Q-*value < 0.05; Table 1). Ten DEGs were enriched in the plant-pathogen interaction pathway under the NHN treatment. When the NHN treatment was compared with the N treatment, calcium-dependent protein kinase 1 (CDPK, TCONS_00113300), heat shock cognate protein 80 (Hsp80, TR82962|c0_g1_i1), calcium-binding protein CML31 (CaMCML31, TCONS_00079635) and disease resistance protein RPS2 (RPS2, TR216110|c0_g1_i2) were up-regulated by 11.6, 2.5, 9.3, and 4.0-fold, respectively. When the NHN treatment was compared with the H treatment, CDPK, Hsp80, CML31, and RPS2 were up-regulated by 2.4, 3.7, 5.8, and 1.7-fold, respectively. Those 1395 DEGs under the NHN treatment were enriched based on the GO database (*P* < 0.05), and included 9, 25, and 20 terms for cellular component, molecular function and biological process categories, respectively (Table S6). GO enrichment results showed that HTSP-related DEGs were mainly involved in membrane proteins, ribonucleoside binding proteins, protein kinase activity, serine family amino acid metabolic processes, phosphotransferase activity, oxylipin metabolism and cell surface receptor signaling pathway processes.

**Table 1:** Significant KEGG pathways under normal-higher-normal (HNH) treatment for 24 h.

**Type**	**Pathway**	**DEGs with pathway annotation**	**All genes with pathway annotation**	***P*-value**	***Q*-value**	**Pathway ID**
NHN (I^*^T)	Ribosome	39 (17.89%)	2309 (4.38%)	7.50E-66	7.28E-64	ko03010
	Plant-pathogen interaction	10 (4.59%)	619 (1.17%)	0.000391	0.0190	ko04626
	Glycerolipid metabolism	7 (3.21%)	392 (0.74%)	0.001317	0.0329	ko00561

*The values of correct-p (Q-value) < 0.05 are considered*.

### Putative *R* genes and TFs involved in HTSP resistance to *Pst*

**Figure 5:**
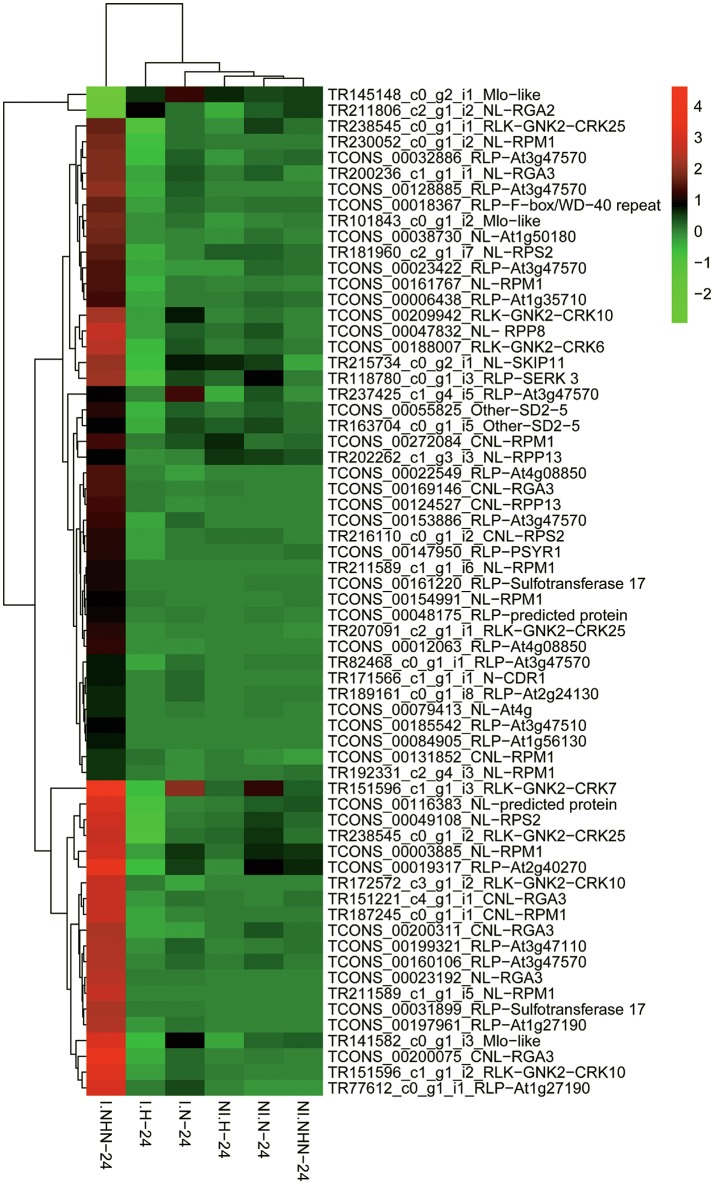
Hierarchical clustering of 64 putative R proteins encoded by differentially expressed genes (DEGs) from the Table 2. The signal ratios are shown in a red-green color scale, where red represents up-regulation and green represents down-regulation. Each column represents the mean expression value (log_2_TMM-FPKM, 24 h sample are divided by values of 0 h samples) of the RNA-Seq data obtained from three biological replicates. Each row represents a DEG.

Sixty-four putative *R* genes (paralogs and spliceforms) mainly belonging to the RLP (23, eLRR-TM-S/TPK domain), NL (18, NBS-LRR domain), and CNL (9, NB-ARC domain) classes were identified (Table 2). The relative expression levels of 58 putative *R* genes were higher in the NHN than in the other treatments (Figure 5). Products of nine, six, three, and two putative *R* gene were homologous to the RPM1, RGA3, RPP13, and RPS2 proteins of *A*. *thaliana*, respectively. Twelve putative *R* gene products were homologous to the LRR receptor-like serine/threonine protein kinase (Ser/Thr PK) of *Aegilops tauschii*, which belong to RLP. Approximately 21% of those up-regulated putative *R* genes were located on B chromosomes (Table 2).

**Table 2:** Summary of predicted HTSP response associated *R* genes homologous under normal-higher-normal (HNH) treatment for 24 h.

**Transcript ID**	**Type**	***E*-value**	**Fold change (NHN vs. N)**	**Fold change (NHN vs. H)**	**Nr functional annotation**	**Chromosome location**
TCONS_00131852	CNL	0	2.818552	2.703682	Disease resistance protein RPM1 [*Aegilops tauschii*]	5DL
TCONS_00124527	CNL	3E-120	3.800193	4.137525	Disease resistance protein RPP13 [*Triticum urartu*]	
TCONS_00200075	CNL	0	7.181612	6.031909	Disease resistance protein RGA3 [*Brachypodium distachyon*]	
TCONS_00200311	CNL	0	5.330094	3.185791	Disease resistance protein RGA3 [*Brachypodium distachyon*]	
TR151221_c4_g1_i1	CNL	0	5.715421	5.109299	Disease resistance protein RGA3 [*Brachypodium distachyon*]	
TCONS_00272084	CNL	0	1.029699	0.62448	Disease resistance protein RPM1 [*Aegilops tauschii*]	7DS
TCONS_00169146	CNL	4E-95	−1.76117	7.233904	Disease resistance protein RGA3 [*Aegilops tauschii*]	
TR216110_c0_g1_i2	CNL	0	4.02049	1.720958	Disease resistance protein RPS2 [*Triticum urartu*]	
TR187245_c0_g1_i1	CNL	2E-38	6.77577	6.946225	Disease resistance protein RPM1 [*Triticum urartu*]	
TR141582_c0_g1_i3	Mlo-like	5E-25	5.27466	4.725616	MLO protein homolog 1	4DL
TR145148_c0_g2_i1	Mlo-like	1E-166	−4.37887	−5.69556	Predicted protein [*Hordeum vulgare* subsp*. vulgare*]	2DS
TR101843_c0_g1_i2	Mlo-like	4E-81	5.305871	4.665338	MLO protein-1-like protein [*Aegilops tauschii*]	4DL
TR202262_c1_g3_i3	NL	0	1.866966	0.266813	Disease resistance protein RPP13 [*Aegilops tauschii*]	6BL
TCONS_00023192	NL	8E-99	7.252431	5.384763	Disease resistance protein RGA3 [*Brachypodium distachyon*]	
TR200236_c1_g1_i1	NL	0	4.637216	3.008804	Disease resistance protein RGA3 [*Brachypodium distachyon*]	
TR211806_c2_g1_i2	NL	0	−5.03394	−5.63732	Disease resistance protein RGA2 [*Triticum urartu*]	2AL
TR215734_c0_g2_i1	NL	9E-73	3.415211	2.013215	Predicted protein [*Hordeum vulgare* subsp*. vulgare*]	4AS
TCONS_00047832	NL	0	3.178319	3.785673	Disease resistance RPP13-like protein 4 [*Aegilops tauschii*]	
TCONS_00049108	NL	0	6.898312	1.918458	Disease resistance protein RPS2 [*Aegilops tauschii*]	2DL
TR181960_c2_g1_i7	NL	0	4.130234	1.477969	Resistance protein [*Triticum aestivum*]	2BL
TCONS_00154991	NL	4E-146	3.296805	3.403045	Disease resistance protein RPM1 [*Triticum urartu*]	7AS
TCONS_00079413	NL	0	2.55712	1.504546	Disease resistance protein At4g27190-like [*Brachypodium distachyon*]	
TR211589_c1_g1_i5	NL	1E-73	−3.12754	12.24328	Disease resistance protein RPM1 [*Aegilops tauschii*]	2DS
TR211589_c1_g1_i6	NL	1E-73	−1.36936	9.992023	Disease resistance protein RPM1 [*Aegilops tauschii*]	2DS
TCONS_00161767	NL	0	4.441952	2.579976	Disease resistance protein RPM1 [*Triticum urartu*]	7BS
TR230052_c0_g1_i2	NL	0	4.911197	2.643646	Disease resistance protein RPM1 [*Triticum urartu*]	7BS
TCONS_00003885	NL	0	2.55409	3.007257	Disease resistance protein RPM1 [*Triticum urartu*]	1AS
TR192331_c2_g4_i3	NL	0	1.397204	1.296923	NBS-LRR disease resistance protein homolog [*Hordeum vulgare*]	7AS
TCONS_00038730	NL	0	5.263287	4.754892	NBS-LRR disease resistance protein homolog [*Hordeum vulgare*]	2BS
TCONS_00116383	NL	1E-35	6.941874	4.123755	Predicted protein [*Hordeum vulgare* subsp*. vulgare*]	5BL
TCONS_00209942	RLK-GNK2	0	3.438375	4.586322	Cysteine-rich receptor-like protein kinase 10 [*Aegilops tauschii*]	3AS
TR172572_c3_g1_i2	RLK-GNK2	9E-173	4.135715	7.999334	Cysteine-rich receptor-like protein kinase 10 [*Aegilops tauschii*]	3AS
TCONS_00188007	RLK-GNK2	0	7.227389	3.927101	Cysteine-rich receptor-like protein kinase 10 [*Aegilops tauschii*]	1DL
TR151596_c1_g1_i2	RLK-GNK2	2E-143	7.46698	4.99814	Cysteine-rich receptor-like protein kinase 10 [*Aegilops tauschii*]	1DL
TR151596_c1_g1_i3	RLK-GNK2	0	7.686276	4.382317	Predicted protein [*Hordeum vulgare* subsp*. vulgare*]	1DL
TR238545_c0_g1_i2	RLK-GNK2	0	6.931735	3.217689	Cysteine-rich receptor-like protein kinase 25 [*Aegilops tauschii*]	1DL
TR207091_c2_g1_i1	RLK-GNK2	2E-153	1.643478	5.98336	Predicted protein [*Hordeum vulgare* subsp*. vulgare*]	
TR238545_c0_g1_i1	RLK-GNK2	0	5.823815	3.253725	Cysteine-rich receptor-like protein kinase 25 [*Aegilops tauschii*]	1DL
TCONS_00128885	RLP	6E-70	6.362442	4.4303	LRR receptor-like serine/threonine-protein kinase [*Aegilops tauschii*]	
TCONS_00012063	RLP	0	4.023625	7.780404	LRR receptor-like serine/threonine-protein kinase [*Aegilops tauschii*]	1BL
TCONS_00022549	RLP	0	3.748471	5.147999	LRR receptor-like serine/threonine-protein kinase [*Aegilops tauschii*]	1DL
TCONS_00185542	RLP	0	2.675208	6.200027	LRR receptor-like serine/threonine-protein kinase [*Aegilops tauschii*]	
TCONS_00160106	RLP	0	6.053778	8.606126	Probable LRR receptor-like serine/threonine-protein kinase [*Brachypodium distachyon*]	7AL
TCONS_00048175	RLP	7E-162	3.243099	4.127629	Unnamed protein product [*Triticum aestivum*]	
TCONS_00199321	RLP	0	4.359545	5.702405	LRR receptor-like serine/threonine-protein kinase [*Aegilops tauschii*]	2BS
TCONS_00032886	RLP	0	3.010608	3.337234	LRR receptor-like serine/threonine-protein kinase [*Aegilops tauschii*]	2BS
TR189161_c0_g1_i8	RLP	0	1.329894	2.968987	LRR receptor-like serine/threonine-protein kinase EFR [*Aegilops tauschii*]	5BL
TCONS_00023422	RLP	0	2.083794	3.047996	LRR receptor-like serine/threonine-protein kinase [*Aegilops tauschii*]	2AS
TCONS_00018367	RLP	3E-32	2.83137	3.420183	F-box/WD-40 repeat-containing protein [*Aegilops tauschii*]	
TCONS_00153886	RLP	0	2.703369	3.923887	LRR receptor-like serine/threonine-protein kinase [*Aegilops tauschii*]	6DL
TR237425_c1_g4_i5	RLP	0	−3.33041	5.691968	LRR receptor-like serine/threonine-protein kinase [*Aegilops tauschii*]	2BL
TCONS_00084905	RLP	0	3.093912	8.190439	Predicted protein [*Hordeum vulgare* subsp. *vulgare*]	3DL
TCONS_00197961	RLP	6.00E-165	6.101454	4.94249	Probable inactive receptor kinase At1g27190 [*Brachypodium distachyon*]	2BL
TR77612_c0_g1_i1	RLP	2.00E-52	4.406603	4.290932	Probable inactive receptor kinase At1g27190 [*Brachypodium distachyon*]	2AL
TR82468_c0_g1_i1	RLP	7.00E-124	2.351209	3.951302	LRR receptor-like serine/threonine-protein kinase At3g47570 [*Oryza brachyantha*]	4BL
TCONS_00147950	RLP	0	3.837951	3.667534	Tyrosine-sulfated glycopeptide receptor 1 [*Aegilops tauschii*]	6DS
TCONS_00031899	RLP	2E-73	7.067972	6.183877	Sulfotransferase 17 [*Aegilop stauschii*]	
TCONS_00161220	RLP	6E-88	3.897112	6.287682	Sulfotransferase 17 [*Aegilops tauschii*]	
TR118780_c0_g1_i3	RLP	6E-38	3.485217	3.081509	Somatic embryogenesis receptor kinase 3, partial [*Commiphora wightii*]	
TCONS_00006438	RLP	0	4.191838	2.755574	LRR receptor-like serine/threonine-protein kinase [*Aegilops tauschii*]	1AL
TR163704_c0_g1_i5	Other	0	3	2.312676	G-type lectin S-receptor-like serine/threonine-protein kinase SD2-5 [*Triticum urartu*]	
TCONS_00019317	RLP	0	8.087051	3.460008	LRR receptor-like serine/threonine-protein kinase [*Aegilops tauschii*]	1DL
TCONS_00055825	Other	0	3.562162	2.970888	Zeamatin [*Aegilops tauschii*]	3AS
TR171566_c1_g1_i1	N	4E-111	1.951928	5.056486	ABC transporter, ATP-binding protein [*Galdieria sulphuraria*]	

*Mlo-like, mlo-like resistant proteins; CNL, contains a central nucleotide-binding (NB) subdomain as part of a larger entity called the NB-ARC domain; RLP, receptor like proteins, consist of a LRR-like repeat, a transmembrane region of ~25 AA, and a short cytoplasmic region, with no kinase domain; NL, contains NBS at N-terminal and LRR at C-terminal, and lacks the CC domain; RLK, class with additional domain GNK2; N, contains NBS domain only; Other, has resistance function but does not fit the known classes. The E-values are calculated by plant resistance gene database. Chromosome location information is from support data 1 and the missing localization information is not found*.

**Figure 6:**
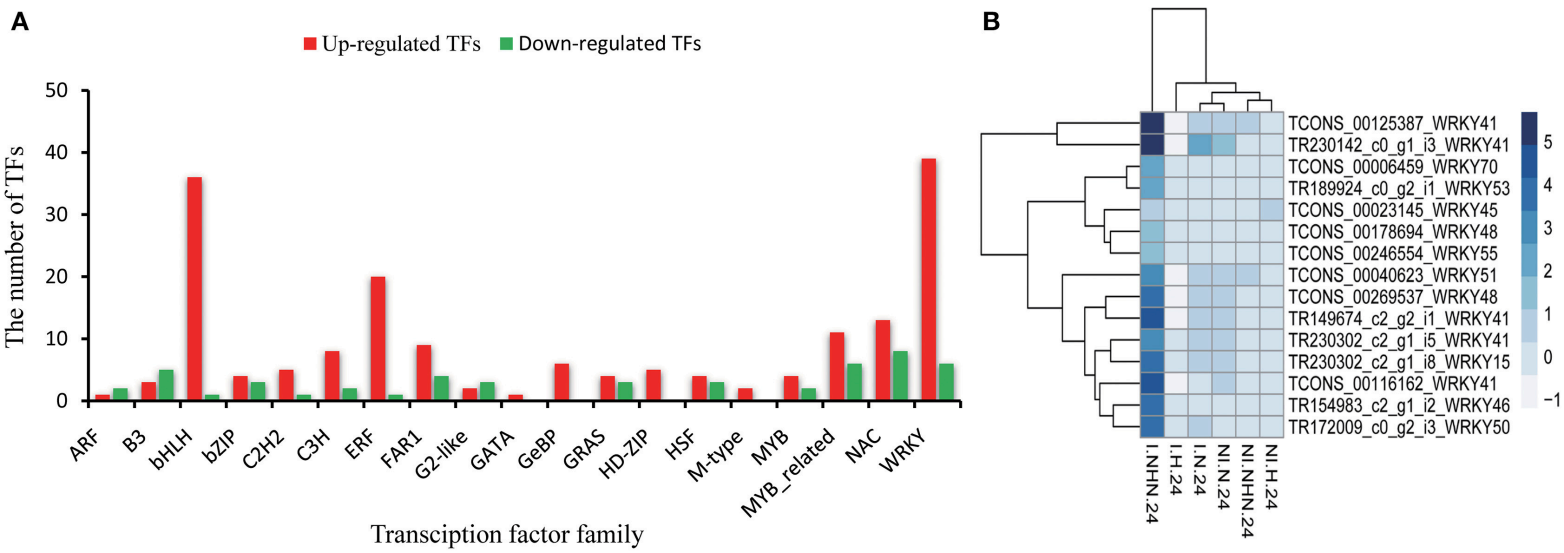
Identification of differentially expressed genes (DEGs) by plant transcription factor database. **(A)** Identification of the distribution feature of differentially expressed transcription factors under the NHN treatment. Red bars represent up-regulated genes and green bars represent down-regulated genes. **(B)** Heatmap of the confirmed WRKY transcription factors (containing complete WRKY domain) responding to HTSP from the NHN treatment.

There were 227 DEGs that were putatively identified as TFs belonging to different families (WRKY, NAC, and MYB, etc.): 177 were up-regulated and 50 were down-regulated in the NHN treatment when compared with the N treatment (Figure 6A). Among these TFs, *WRKY* was the family with the largest number of genes, but further domain alignment analysis indicated that only 17 (including alternative splicing) of these genes contained a complete WRKY domain and the zinc finger motif type, including *WRKY41, WRKY70, WRKY55, WRKY53, WRKY51, WRKY50, WRKY48, WRKY46, WRKY45* and *WRKY15*; most of these *WRKY* genes were up-regulated under the NHN treatment compared with those under the other treatments. Among the *WRKY* genes, the relative expression level of *WRKY 41* was the highest; it had five homologs (paralogs or spliceforms) under the NHN treatment (Figure 6B).

### Protein interaction network in the HTSP response to *Pst*

**Figure 7:**
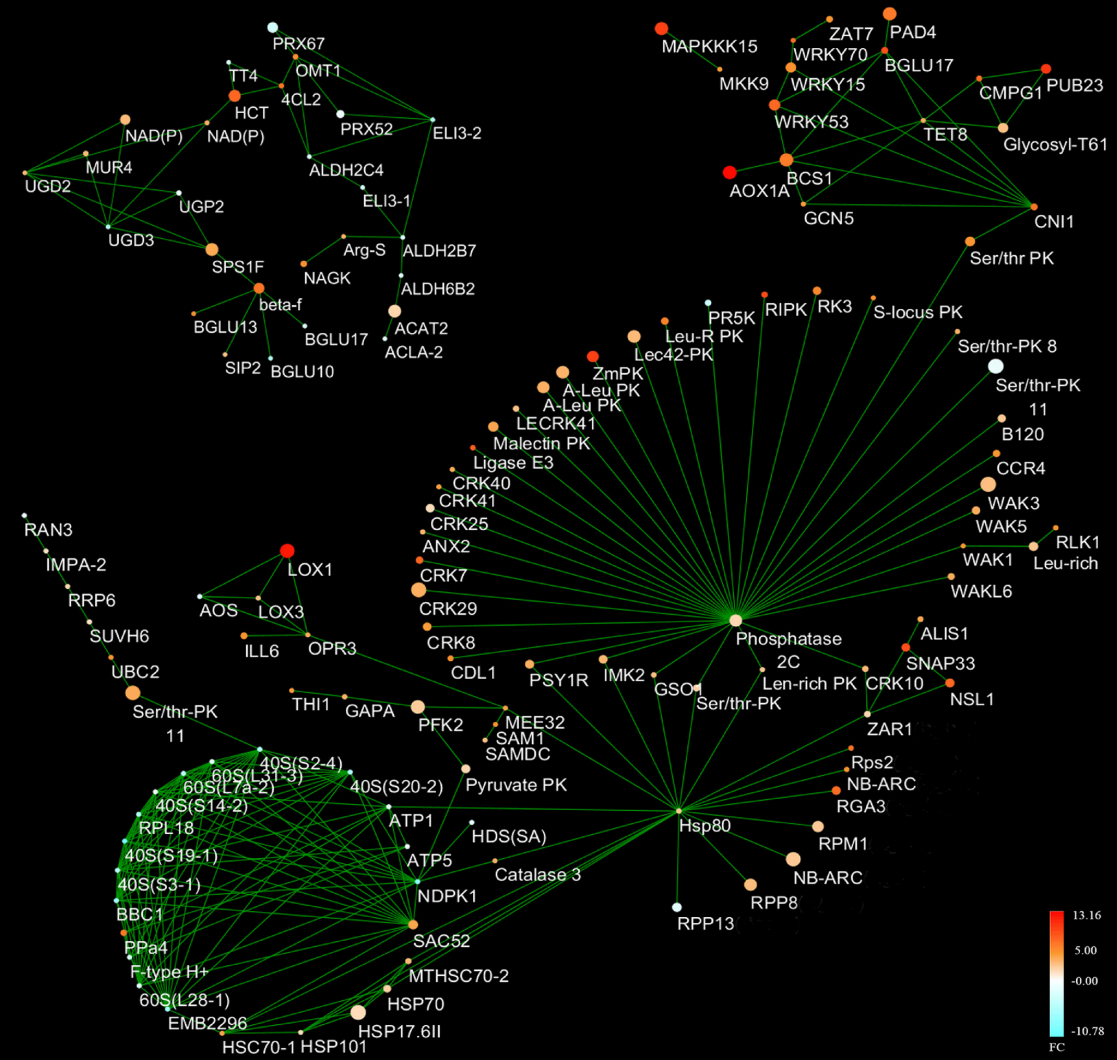
Protein interaction network in higher-temperature seedling-plant (HTSP) responding to *Puccinia striiformis* f. sp. *tritici* under the normal-higher-normal (NHN) temperature treatment. Different colors represent fold changes of differentially expressed genes (DEGs) (NHN vs. N). Each node is a DEG. Each size of the node represents a false discover rate (FDR) value and the smaller node was more significant than a bigger one. The FDR values ranged from 1.39E-59 to 0.049924. Protein interaction network was constructed by CYTOSCAPE software.

A total of 135 proteins, mostly consisting of lignin and fatty acid synthesis related proteins, ribosome proteins, protein kinases, heat shock proteins, WRKY TFs, and R proteins homologous, were involved in the main network based on the STRING database of *A. thaliana* (Data S2; Figure 7). In particular, compared to other proteins, phosphatase 2C10 (PP2C10, TCONS_00197067) was the hub protein with the highest degree of 52 (Data S2); this protein was predicted to interact with 35 protein kinases. In addition, most of R proteins interacted with Hsp80. For example, Hsp80 interacted with Ser/Thr PK and RPS2. Additionally, lignin and fatty acid biosynthesis related proteins formed a separate interaction network. For instance, 4-coumarate ligase 2 (4CL2, TR211614|c0_g1_i10), elicitor-activated gene 3-1 (ELI3-1, TR172862|c0_g1_i2), and elicitor-activated gene 3-2 (ELI3-2, TR171566|c1_g1_i1) were associated with lignin biosynthesis. When the NHN treatment was compared with both the N and H treatments, the majority of the ribonucleoproteins (RPs) encoded by DEGs interacted with each other and all these DEGs were down-regulated.

## Discussion

### Identification of differentially expressed genes under the NHN treatment involved in HTSP resistance to *Pst*

Studies on the inheritance and ultrastructural analysis of the HTSP resistance in XY 6 have been reported previously (Wang and Shang, 2003; Yao et al., 2006). These studies showed that constant relatively high temperature cannot induce resistance to *Pst* in XY 6 (Shang and Wang, 1997) and that only temperature changes can activate the resistance. Similarly, HTAP resistance to *Pst* becomes effective when wheat plants were grown under a night/day cycle of 10~12°C/25~30°C after inoculation (Chen, 2005, 2013; Coram et al., 2008a; Bryant et al., 2014). In fact, wheat crops grown in the fields are almost all under fluctuating temperature conditions every day and changing temperatures throughout the growth season, and therefore, screening germplasm for stripe rust resistance should be carried out at diurnally changed temperatures (Chen, 2013). Wheat plants at 18°C facilitated resistance to the *Triticum mosaic virus* (TriMV), which was controlled by temperature dependent *Wsm1* and *Wsm2* genes (Tatineni et al., 2016). The present study confirmed that the HTSP resistance in XY 6 was activated by exposure to 20°C for 24 h. Also, the expression level trends of 1395 DEGs in both the H and N treatments were similar, consistent with the histological data on *Pst* development. These results showed that sudden changes in temperature play an active role in the defense response to *Pst* in XY 6. Coram et al. (2008a) identified 99 transcripts involved in the *Yr39*-mediated HTAP resistance to stripe rust, including R protein homologs, pathogenesis-related (PR) proteins, protein kinases and phenylpropanoid biosynthesis. In contrast, we identified a total of 1395 DEGs in the HTSP resistance induced by the NHN treatment, including genes coding phosphatase 2C10, protein kinases, R protein homologs, TFs and RPs as well function unknown proteins were specific resistance. The high number of DEGs was achieved by taking the advantage of RNA-Seq over the previously used microarray technique (Coram et al., 2008a).

### Phosphatase 2C proteins may play a positive role in HTSP resistance to *Pst*

Phosphatase 2C (PP2C) has been reported to be involved in the regulation of plant development and the adaptation to environmental stresses (Schweighofer et al., 2004; Bhatnagar et al., 2017). Recently, there has been an increasing focus on the role of PP2C in plant stress signaling: cold, drought, high salt, etc. (Hu et al., 2010; Liu et al., 2012; Arshad and Mattsson, 2014), indicating that PP2C could receive signals rapidly under abiotic stresses. However, reports about the function of PP2C in disease resistance are limited. In an alien substitution line of *Triticum aestivum-Elytrigia elongatum*, a phosphateserine aminotransferase was shown to be involved in defense to powdery mildew (He and Wang, 2005). Protein kinases such as Ser/Thr PK can alter the functions of proteins by phosphorylating the OH group of serine or threonine residues, and protein phosphorylation plays an important role in disease resistance (Cao et al., 2011). In the present study, PP2C10 in XY 6 was up-regulated and predicted to directly interact with 35 protein kinases during the induction process of HTSP resistance based on the analysis of the database of *Arabidopsis* protein interactions. This suggests that PP2C10 could be a hub protein and may play a pivotal role, such as signal switch in HTSP resistance against *Pst* by regulating the activity of protein kinases.

### Chromosomal locations and predicted functions of *R* genes

Wang and Chen (2017) summarized a total of 451 genes and QTL with chromosomal locations for resistance to stripe rust in wheat identified through molecular mapping, of which 49% are on B chromosomes while only 31% on A chromosomes and 20% on D chromosomes, indicating that the B genome is more involved in stripe rust resistance than either A or D genomes. In the present study, we found 41% of the 3731 DEGs with mapped chromosomal locations were on B chromosomes, more than either A chromosomes (27.2%) or D chromosomes (29.7%). Furthermore, 21% of the 64 *R* genes were on B chromosomes. Our results also suggest that the B genome of wheat contains more genes for defense to *Pst*.

There have been many studies on temperature sensitive *R* genes against stripe rust, especially non-race-specific HTAP resistance, such as *Yr36* (Fu et al., 2009; Bryant et al., 2014); *Yr52* (Ren et al., 2012); *Yr59* (Zhou et al., 2014), *Yr62* (Lu et al., 2014), *LrZH22* (Wang et al., 2016), and *Yr79* (Feng et al., 2018). Previously cloned non-race-specific resistance genes to stripe rust and/or leaf rust, such as *Yr18/Lr34* (Krattinger et al., 2009a), *Yr36* (Fu et al., 2009), and *Yr46/Lr67* (Moore et al., 2015), do not contain NBS-LRR domains (Chen, 2013), in contrast to many race-specific resistance to stripe rust and leaf rust, such as *Lr10* (Feuillet et al., 2003), *Lr21* (Huang et al., 2003); *Lr1* (Cloutier et al., 2007); *Yr10* (Liu W. et al., 2014). Base on the association of the types of genes to the types of resistance, temperature-sensitive and non-NBS-LRR genes have been connected to the non-race specificity and therefore durability of stripe rust resistance (Chen, 2013; Chen et al., 2013; Wang and Chen, 2017). Although we did not study genes in XY 6 for controlling the HTSP resistance to stripe rust as in those studies mentioned above, we identified 23 putative *R* genes encoding an eLRR-TM-S/TPK domain and most of them were up-regulated during the HTSP induction process. The finding of the numerous *R* genes homologs involved in HTSP resistance is similar to nine *R* genes contributing to the *Yr39*-controlled HTAP resistance (Coram et al., 2008a). The LRR domain has been implicated in PPIs (Martin et al., 2003). PPIs related to disease resistance can be complex. For example, the RPM1 protein of *A. thaliana* contains NBS and LRR domains, and RPM1-interacting protein 4 (RIN4), a membrane receptor protein, recognizes the *avrRpt2* effector (Axtell and Staskawicz, 2003). The cleavage of RIN4 results in *RPS2*-mediated elicitor-triggered immunity, which can be induced by relatively high temperature (Wang et al., 2009; Zhu et al., 2010). It will be interesting to further study the putative *R* genes identified in the present study to determine how they interact to each other and to other genes contributing to the HTSP resistance.

Hsp80, which is involved in a variety of regulatory and defense responses, has a molecular chaperone function in which the protein interacts with different domains of R proteins (Takahashi et al., 2003; Maimbo et al., 2007; Wang et al., 2011). The present study predicts that Hsp80 interacts with different domains of R proteins based on the analysis of the database of *Arabidopsis* protein interactions, such as the NBS-LRR (RPM1, RPS2) and eLRR-TM-S/TPK (Serine threonine-protein kinase) domains. Moreover, the Pto and PBS1 proteins are members of the R protein family containing a S/TPK domain and have been shown to require the NBS-LRR-domain R proteins Prf and RPS5 respectively for their resistance functions (Brueggeman et al., 2008). Based on the up-regulation of Hsp80 and putative *R* genes during the HTSP induction, we hypothesize that the eLRR-TM-S/TPK domains of the putative R proteins could interact with the NBS-LRR-domain proteins via the Hsp80 protein, leading to the HTSP resistance in XY 6 against *Pst*.

### CDPK and ribosomal proteins are associated with important Ca^2+^ signaling components involved in HTSP

The CDPK gene was highly up-regulated in the induction process of HTSP. CDPK is an important protein associated with Ca^2+^ signaling components in immune and stress signaling networks (Bolton, 2009). Previous reports have shown that calcium-dependent CDPK4 and CDPK5 regulate ROS production by phosphorylating NADPH oxidase in potato (Kobayashi et al., 2007). However, ROS is important not only for signaling mechanisms for defense (Eckardt, 2017) but also for regulating programmed cell death through the establishment of the HR (Tamas et al., 2010). Therefore, the CDPK protein identified in the present study may function in ROS accumulation and cell death under HTSP resistance against *Pst*. In addition, a ribosome translocon complex mediates calcium leakage from endoplasmic reticulum stores, which regulate many physiological processes, including apoptosis (Coppenolle et al., 2004; Garcia et al., 2017). Hence, we suggest that during the initial stage of higher temperature treatment, the down-regulation of ribosomal genes in XY 6 may be part of an emergency reaction against temperature stress. This process could change the composition of ribosomes (Wang et al., 2013), involved in the regulation of calcium leakage, and increase resistance to *Pst* in XY 6.

### WRKY TFs are positively involved in the HTSP resistance of XY 6 to *Pst*

WRKY TFs belong to a large gene family, are regulated by MAPKs and mediate plant defense responses (Chen et al., 2012). A large number of studies have shown that most of WRKY TFs are involved in the salicylic acid (SA) signaling pathway in defense responses (Dong et al., 2003; Xing et al., 2008; Hu Y. et al., 2012; Shimono et al., 2012; Wang et al., 2017). A putative *WRKY5* gene was found to be up-regulated in the HTAP resistance controlled by *Yr39* (Coram et al., 2008a). In the present study, 17 *WRKY* genes, including *WRKY41* and *WRKY70*, were identified to be up-regulated during the induction process of HTSP resistance. Overexpression of *WRKY41* and *WRKY70* leads to the constitutive expression of *PR5* and *PR1* genes in *A*. *thaliana*, which increased resistance to *P. syringae* pv. *syringae* and *Erysiphe cichoracearum*, respectively (Li et al., 2006; Higashi et al., 2008). Silencing *TaWRKY70* leads to enhanced susceptibility to *Pst* when subjected to higher temperature during the initial *Pst* incubation stage (Wang et al., 2017). Moreover, *WRKY* proteins interact with not only PR proteins and receptor-like kinases but also other members of the WRKY family in disease resistance (Yu et al., 2001; Peng et al., 2008; Hu Y. et al., 2012). Based on the criterion that PPI confidence scores were greater than 0.7, the interactions between WRKY15 (FDR < 0.02) and WRKY70 (FDR < 0.00002), WRKY15 (FDR < 0.02) and WRKY53 (FDR < 0.03) were identified. These results indicate that WRKY TFs may work together with other members of WRKY proteins and play an important role in the crosstalk of SA signaling pathways in HTSP resistance to *Pst*.

### DEGs are involved in signaling pathways during the HTSP response of XY 6 to *Pst*

**Figure 8:**
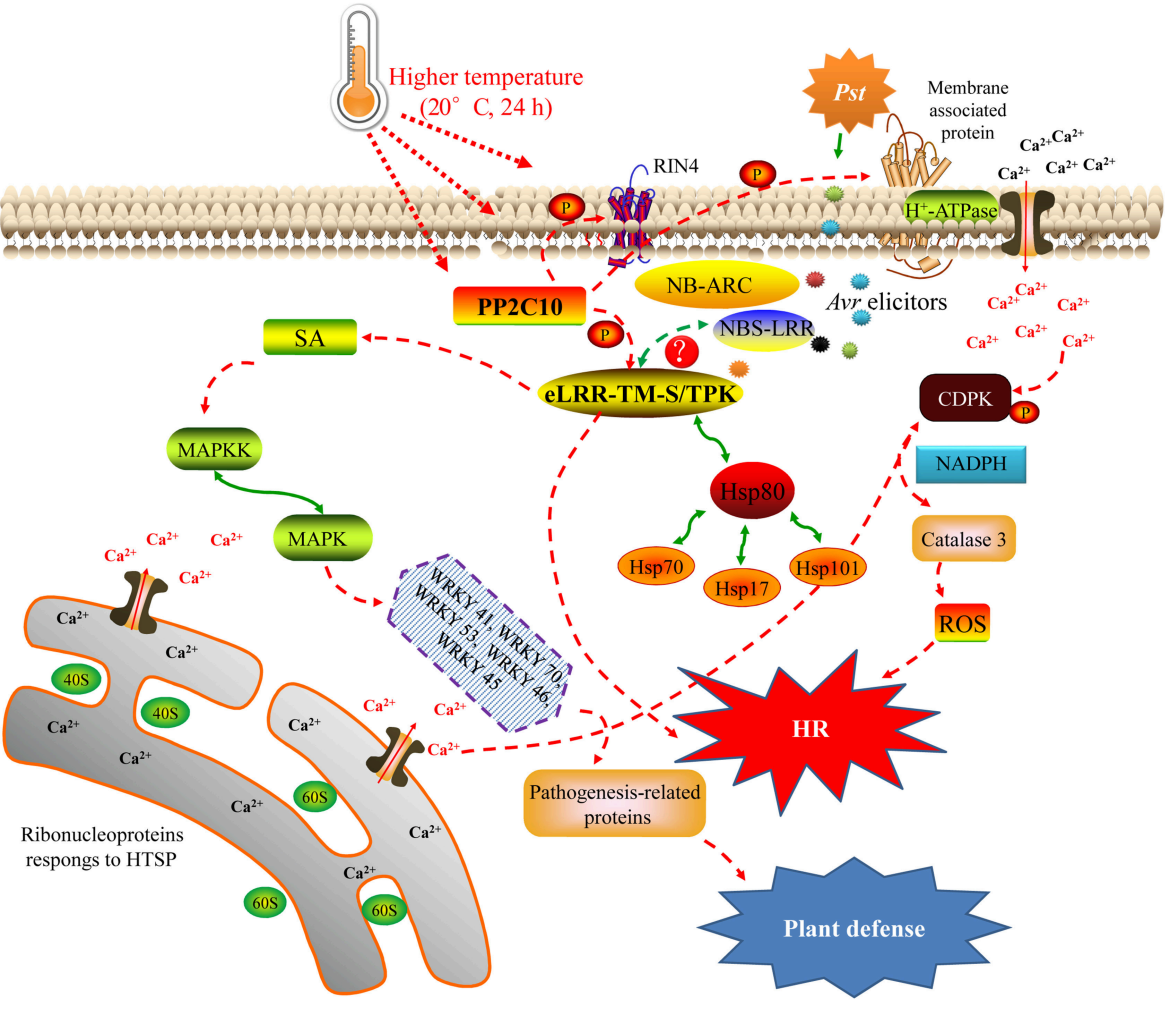
A summary of the molecular pathways and cellular processes in higher-temperature seedling-plant (HTSP) resistance to *Puccinia striiformis* f. sp. *tritici* in XY 6. The red dotted lines indicate the proposed pathways and the green lines denote the protein-protein interactions based on the KEGG and PPIs database of *Arabidopsis thaliana*. A question mark indicates the interaction of proteins that needs functional verification.

A complex network of signaling pathways induced by phytohormones such as SA regulates local and systemic resistance to invasive pathogens (Panstruga et al., 2009). We identified several major signal transduction pathways that are likely involved in the HTSP response of XY 6 to *Pst* (Figure 8). First, HTSP resistance to *Pst* is induced by temperature changes and *Pst* inoculation, which may induce changes in the phosphorylation status of Ser/Thr PK via PP2C10 or membrane associated proteins such as RIN4. These changes could then lead to *R* genes directly or indirectly recognizing *Avr* elicitors of *Pst* by interacting with Ser/Thr PK or RIN4, activating the HR of XY 6 with the help of Hsp proteins. Second, *R* gene-mediated resistance pathways may activate SA signaling pathways, which regulate downstream MAPK proteins and transcription factors (WRKY); this process results in a defense response in XY 6. Third, phosphorylation of membrane-associated proteins may activate H^+^-ATPases and act as an important primary sensor of Ca^2+^ leakage, leading to the activation of CDPK or RPs. This process would subsequently regulate ROS production by phosphorylating NADPH oxidase, leading to the HR in XY 6.

In summary, HTSP resistance to *Pst* in XY 6 is induced by the exposure to 20°C for 24 h during the early *Pst* incubation stage and may be controlled by several major genes in conjunction with other minor-effect genes. Functions of many identified DEGs remain unknown although some candidate genes were identified based on the current NCBI database. For example, PP2C10 and LRR receptor-like serine/threonine protein kinases may play important roles in the processes of SA and Ca^2+^ signal transduction in HTSP resistance to *Pst*. Identified DEGs were located on A chromosomes (29.2%), B chromosomes (41.1%), and D chromosomes (29.7%); most of the defense related DEGs were located on the B chromosome group. Together, these results constitute a strong base for future research on HTSP resistance to *Pst* in wheat.

## Author contributions

XH, XX, and JY planned and designed the research. FT, JW, and ZG performed RNA-Seq experiments. FT and JH analyzed the data. FT, XH, XC, and XX wrote the manuscript.

### Conflict of interest statement

The authors declare that the research was conducted in the absence of any commercial or financial relationships that could be construed as a potential conflict of interest.

## Supplementary material

Figure S1 The experiment processes of three different temperature treatments. The red lines indicate the higher temperature (20°C) treatment. The black lines indicate the normal temperature (15°C) treatment. The 8~9 days post-inoculation (dpi) are equivalent to 192~216 h after inoculation. The dotted lines indicate the samples at 0 and 24 h (9 dpi) of XY 6 for RNA-Seq. N treatment: constant normal temperature (15°C); NHN treatment: switch of normal and higher temperatures (15°C~20°C~15°C); and H: constant higher temperature (20°C). CK: sterile water-inoculated wheat plants used as control for all treatments.

Figure S2 Amplification efficiency and melting curve of each transcripts. ATP-dependent 26S proteasome regulatory subunit (26S, black) and cell division control (CDC, red) proteins are chosen as reference genes. The target gene is indicated in blue.

Figure S3 Sequences **(A)** and open reading form (ORF) **(B)** length distribution of the RNA-Seq data using the CDMC assembly.

Table S1 Kolmogorov-Smirnov test for each treated sample.

Table S2 List of primers used in qRT-PCR.

Table S3 Q30 quality levels of 30 RNA-Seq samples.

Table S4 Statistics of *de novo*, mapping and CDMC assembly strategies.

Table S5 Statistical table of differentially expressed genes (DEGs) and annotated DEGs from the CDMC assembly.

Table S6 Go characterization of differentially expressed genes (DEGs) responding to *Puccinia striiformis* f. sp. *tritici* under the normal-higher-normal (NHN) temperature treatment.

Data S1 Chromosome location and annotation of differentially expressed genes (DEGs) in normal-higher-normal (NHN) temperature treatment vs. normal (N) (I^*^T) and NHN vs. higher (H) (I^*^T) temperature treatments.

Data S2 Protein interaction network derived from differentially expressed genes (DEGs) in higher-temperature seedling-plant (HTSP) resistance to *Puccinia striiformis* f. sp. *tritici*.

## References

[B1] An F. Tao F. Wang J. J. Tian W. Shang H. S. Hu X. P. (2015). Optimal conditions of expression of high-temperature resistance to stripe rust in Xiaoyan 6. J. Triticeae Crops 35, 1314–1319.

[B2] Arshad M. Mattsson J. (2014). A putative poplar PP2C-encoding gene negatively regulates drought and abscisic acid responses in transgenic *Arabidopsis thaliana*. Trees Struct. Funct. 28, 531–543. 10.1007/s00468-013-0969-7

[B3] Axtell M. J. Staskawicz B. J. (2003). Initiation of *RPS2*-specified disease resistance in Arabidopsis is coupled to the AvrRpt2-directed elimination of RIN4. Cell. 112, 369–377. 10.1016/S0092-8674(03)00036-9 12581526

[B4] Benjamini Y. Hochberg Y. (1995). Controlling the false discovery rate: a practical and powerful approach to multiple testing. J. R. Stat. Soc. 57, 289–300

[B5] Bhatnagar N. Min M. K. Choi E. H. Kim N. Moon S. J. Yoon I.. (2017). The protein phosphatase 2C clade A protein OsPP2C51 positively regulates seed germination by directly inactivating OsbZIP10. Plant Mol. Biol. 93, 389–401. 10.1007/s11103-016-0568-2 28000033

[B6] Bolger A. M. Lohse M. Usadel B. (2014). Trimmomatic: a flexible trimmer for Illumina sequence data. Bioinformatics 30, 2114–2120. 10.1093/bioinformatics/btu170 24695404 PMC4103590

[B7] Bolton M. D. (2009). Primary metabolism and plant defense-fuel for the fire. Mol. Plant Microbe Interact. 22, 487–497. 10.1094/MPMI-22-5-0487 19348567

[B8] Brueggeman R. Druka A. Nirmala J. Cavileer T. Drader T. Rostoks N.. (2008). The stem rust resistance gene *Rpg5* encodes a protein with nucleotide-binding-site, leucine-rich, and protein kinase domains. Proc. Natl. Acad. Sci. U.S.A. 105, 14970–14975. 10.1073/pnas.0807270105 18812501 PMC2567477

[B9] Bryant R. R. McGrann G. R. Mitchell A. R. Schoonbeek H. J. Boyd L. A. Uauy C.. (2014). A change in temperature modulates defence to yellow (stripe) rust in wheat line UC1041 independently of resistance gene *Yr36*. BMC Plant Biol. 14:10. 10.1186/1471-2229-14-10 24397376 PMC3898064

[B10] Cao A. Xing L. Wang X. Yang X. Wang W. Sun Y.. (2011). Serine/threonine kinase gene *Stpk-V*, a key member of powdery mildew resistance gene *Pm21*, confers powdery mildew resistance in wheat. Proc. Natl. Acad. Sci. U.S.A. 108, 7727–7732. 10.1073/pnas.1016981108 21508323 PMC3093467

[B11] Chen L. Song Y. Li S. Zhang L. Zou C. Yu D. (2012). The role of WRKY transcription factors in plant abiotic stresses. Biochim. Biophys. Acta 1819, 120–128. 10.1016/j.bbagrm.2011.09.002 21964328

[B12] Chen X. M. (2005). Epidemiology and control of stripe rust (*Puccinia striiformis* f. sp. *tritici)* on wheat. Can. J. Plant Pathol. 27, 314–337. 10.1080/07060660509507230

[B13] Chen X. M. (2007). Challenges and solutions for stripe rust control in the United States. Aust. J. Agr Res. 58, 648–655. 10.1071/AR07045

[B14] Chen X. M. (2013). Review article: high-temperature adult-plant resistance, key for sustainable control of stripe rust. Am. J. Plant Sci. 4, 608–627. 10.4236/ajps.2013.43080

[B15] Chen X. M. Coram T. E. Huang X. L. Wang M. N. Dolezal A. (2013). Understanding molecular mechanisms of durable and non-durable resistance to stripe rust in wheat using a transcriptomics approach. Cur. Genomics 14, 111–126. 10.2174/1389202911314020004 24082821 PMC3637676

[B16] Chen X. M. Line R. F. (1995). Gene action in wheat cultivars for durable, high-temperature, adult-plant resistance and interaction with race-specific, seedling resistance to *Puccinia striiformis*. Phytopathology 85, 567–572. 10.1094/Phyto-85-567

[B17] Chhetri M. Bariana H. Kandiah P. Bansal U. (2016). *Yr58*: a new stripe rust resistance gene and its interaction with *Yr46* for enhanced resistance. Phytopathology 106, 1530–1534. 10.1094/PHYTO-04-16-0182-R 27673348

[B18] Cloutier S. McCallum B. D. Loutre C. Banks T. W. Wicker T. Feuillet C.. (2007). Leaf rust resistance gene *Lr1*, isolated from bread wheat (*Triticum aestivum* L.) is a member of the large psr567 gene family. Plant Mol. Biol. 65, 93–106. 10.1007/s11103-007-9201-8 17611798

[B19] Conesa A. Götz S. Garcíagómez J. M. Terol J. Talón M. Robles M. (2005). Blast2GO: a universal tool for annotation, visualization and analysis in functional genomics research. Bioinformatics 21, 3674–3676. 10.1093/bioinformatics/bti610 16081474

[B20] Coppenolle F. V. Abeele F. V. Slomianny C. Flourakis M. Hesketh J. Dewailly E.. (2004). Ribosome-translocon complex mediates calcium leakage from endoplasmic reticulum stores. J. Cell Sci. 117, 4135–4142. 10.1242/jcs.01274 15280427

[B21] Coram T. E. Huang X. L. Zhan G. M. Settles M. L. Chen X. M. (2010). Meta-analysis of transcripts associated with race-specific resistance to stripe rust in wheat demonstrates common induction of blue copper-binding protein, heat-stress transcription factor, pathogen-induced WIR1A protein, and ent-kaurene synthase transcripts. Funct. Integrat. Genomics 10, 383–392. 10.1007/s10142-009-0148-5 19937262

[B22] Coram T. E. Settles M. L. Chen X. M. (2008a). Transcriptome analysis of high-temperature adult-plant resistance conditioned by *Yr39* during the wheat- *Puccinia striiformis* f. sp. *tritici* interaction. Mol. Plant Pathol. 9, 479–493. 10.1111/j.1364-3703.2008.00476.x 18705862 PMC6640281

[B23] Coram T. E. Wang M. N. Chen X. M. (2008b). Transcriptome analysis of the wheat-*Puccinia striiformis* f. sp. *tritici* interaction. Mol. Plant Pathol. 9, 157–169. 10.1111/j.1364-3703.2007.00453.x 18705849 PMC6640478

[B24] Dong J. Chen C. Chen Z. (2003). Expression profiles of the *Arabidopsis WRKY* gene superfamily during plant defense response. Plant Mol. Biol. 51, 21–37. 10.1023/A:1020780022549 12602888

[B25] Eckardt N. A. (2017). The plant cell reviews plant immunity: receptor-like kinases, ROS-RLK crosstalk, quantitative resistance, and the growth/defense trade-off. Plant Cell 29, 601–602. 10.1105/tpc.17.00289 28396552 PMC5435444

[B26] Feng J. Y. Wang M. N. See D. R. Chao S. M. Zheng Y. L. Chen X. M. (2018). Characterization of novel gene *Yr79* and four additional QTL for all-stage and high-temperature adult-plant resistance to stripe rust in spring wheat PI 182103. Phytopathology. [Epub ahead of print]. 10.1094/PHYTO-11-17-0375-R 29303685

[B27] Feuillet C. Travella S. Stein N. Albar L. Nublat A. Keller B. (2003). Map-based isolation of the leaf rust disease resistance gene *Lr10* from the hexaploid wheat (*Triticum aestivum* L.) genome. Proc. Natl. Acad. Sci. U.S.A. 100, 15253–15258. 10.1073/pnas.2435133100 14645721 PMC299976

[B28] Franceschini A. Szklarczyk D. Frankild S. Kuhn M. Simonovic M. Roth A.. (2013). STRING v9.1: protein-protein interaction networks, with increased coverage and integration. Nucleic Acids Res. 41, 808–815. 10.1093/nar/gks1094 23203871 PMC3531103

[B29] Fu D. Uauy C. Distelfeld A. Blechl A. Epstein L. Chen X.. (2009). A kinase-START gene confers temperature-dependent resistance to wheat stripe rust. Science 323, 1357–1360. 10.1126/science.1166289 19228999 PMC4737487

[B30] Fu L. Niu B. Zhu Z. Wu S. Li W. (2012). CD-HIT: accelerated for clustering the next-generation sequencing data. Bioinformatics 28, 3150–3152. 10.1093/bioinformatics/bts565 23060610 PMC3516142

[B31] Garcia M. I. Chen J. J. Boehning D. (2017). Genetically encoded calcium indicators for studying long-term calcium dynamics during apoptosis. Cell Calcium 61, 44–49. 10.1016/j.ceca.2016.12.010 28073595 PMC5512452

[B32] Haas B. J. Papanicolaou A. Yassour M. Grabherr M. Blood P. D. Bowden J.. (2013). *De novo* transcript sequence reconstruction from RNA-Seq using the Trinity platform for reference generation and analysis. Nat. Protoc. 8, 1494–1512. 10.1038/nprot.2013.084 23845962 PMC3875132

[B33] Hao Y. Wang T. Wang K. Wang X. Fu Y. Huang L.. (2016). Transcriptome analysis provides insights into the mechanisms underlying wheat plant resistance to stripe rust at the adult plant stage. PLoS ONE 11:e0150717. 10.1371/journal.pone.0150717 26991894 PMC4798760

[B34] He D. Y. Wang H. G. (2005). Cloning of a novel phosphateserine aminotransferase gene from a *Triticum aestivum*-*Elytrigiaelongatum* alien substitution line with resistance to powdery mildew. Chin. Sci. Bull. 50, 646–651. 10.1360/982004-445

[B35] Herrera-Foessel S. A. Lagudah E. S. Huerta-Epino J. Hayden M. Bariana H. Singh D. (2011). New slow-rusting leaf rust and stripe rust resistance genes *Lr67* and *Yr46* are pleiotropic or closely linked. Theor. Appl. Genet. 122, 239–249. 10.1007/s00122-010-1439-x 20848270

[B36] Higashi K. Ishiga Y. Inagaki Y. Toyoda K. Shiraishi T. Ichinose Y. (2008). Modulation of defense signal transduction by flagellin-induced WRKY41 transcription factor in *Arabidopsis thaliana*. Mol. Genet. Genomics 279, 303–312. 10.1007/s00438-007-0315-0 18219494

[B37] Hu X. Liu L. Xiao B. Li D. Xing X. Kong X.. (2010). Enhanced tolerance to low temperature in tobacco by over-expression of a new maize protein phosphatase 2C, ZmPP2C2. J. Plant Physiol. 167, 1307–1315. 10.1016/j.jplph.2010.04.014 20580122

[B38] Hu X. P. Wang Y. T. Shang H. S. (2012). Characteristics of expression of high-temperature resistance to stripe rust in Xiaoyan 6. Chin. Acta Agric. Boreali-Occident Sin. 21, 43–47. 10.3969/j.issn.1004-1389.2012.02.009

[B39] Hu Y. Dong Q. Yu D. (2012). *Arabidopsis* WRKY46 coordinates with WRKY70 and WRKY53 in basal resistance against pathogen *pseudomonas syringae*. Plant Sci. 186, 288–297. 10.1016/j.plantsci.2011.12.003 22325892

[B40] Huang L. Brooks S. A. Li W. Fellers J. P. Trick H. N. Gill B. S. (2003). Map-based cloning of leaf rust resistance gene *Lr21* from the large and polyploid genome of bread wheat. Genetics 164, 655–664. 12807786 10.1093/genetics/164.2.655 PMC1462593

[B41] Jin J. Zhang H. Kong L. Gao G. Luo J. (2014). PlantTFDB 3.0: a portal for the functional and evolutionary study of plant transcription factors. Nucleic Acids Res. 42, 1182–1187. 10.1093/nar/gkt1016 24174544 PMC3965000

[B42] Kobayashi M. Ohura I. Kawakita K. Yokota N. Fujiwara M. Shimamoto K.. (2007). Calcium-dependent protein kinases regulate the production of reactive oxygen species by potato NADPH oxidase. Plant Cell 19, 1065–1080. 10.1105/tpc.106.048884 17400895 PMC1867354

[B43] Krattinger S. G. Jordan D. R. Mace E. S. Raghavan C. Luo M. C. Keller B.. (2013). Recent emergence of the wheat *Lr34* multipathogen resistance: insights from haplotype analysis in wheat, rice, sorghum and *Aegilops tauschii*. Theor. Appl. Genet. 126, 663–672. 10.1007/s00122-012-2009-1 23117720

[B44] Krattinger S. G. Lagudah E. S. Spielmeyer W. Singh R. P. Huerta-Espino J. McFadden M.. (2009a). A putative ABC transporter confers durable resistance to multiple fungal pathogens in wheat. Science 323, 1360–1363. 10.1126/science.1166453 19229000

[B45] Krattinger S. G. Lagudah E. S. Wicker T. Risk J. M. Ashton A. R. Selter L. L.. (2011). *Lr34* multi-pathogen resistance ABC transporter: molecular analysis of homoeologous and orthologous genes in hexaploid wheat and other grass species. Plant J. 65, 392–403. 10.1111/j.1365-313X.2010.04430.x 21265893

[B46] Krattinger S. G. Sucher J. Selter L. L. Chauhan H. Zhou B. Tang M.. (2016). The wheat durable, multipathogen resistance gene *Lr34* confers partial blast resistance in rice. Plant Biotechnol. J. 14, 1261–1268. 10.1111/pbi.12491 26471973 PMC11388880

[B47] Krattinger S. G. Wicker T. Keller B. (2009b). Map-based cloning of genes in Triticeae (wheat and barley), in Genetics and Genomics of the Triticeae, Plant Genetics and Genomics: Crops and Models, Vol. 7, eds Feuillet C. Muehlbauer G. J. (New York, N. Y: Springer), 339–358.

[B48] Laudencia-Chingcuanco D. L. Stamova B. S. Lazo G. R. Cui X. Anderson O. D. (2006). Analysis of the wheat endosperm transcriptome. J. Appl. Genet. 47, 287–302. 10.1007/BF03194638 17132893

[B49] Li B. Dewey C. N. (2011). RSEM: accurate transcript quantification from RNA-Seq data with or without a reference genome. BMC Bioinformatics 12:323 10.1186/1471-2105-12-323 21816040 PMC3163565

[B50] Li H. Z. Gao X. Li X. Y. Chen Q. J. Dong J. Zhao W. C. (2013). Evaluation of assembly strategies using RNA-Seq data associated with grain development of wheat (*Triticum aestivum* L.). PLoS ONE 8:e83530. 10.1371/journal.pone.0083530 24349528 PMC3861526

[B51] Li J. Brader G. Kariola T. Palva E. T. (2006). WRKY70 modulates the selection of signaling pathways in plant defense. Plant J. 46, 477–491. 10.1111/j.1365-313X.2006.02712.x 16623907

[B52] Li Z. S. (1986). Distant hybridization of wheat new varieties Xiaoyan 6. Sci. Agric. Sin. 5:30.

[B53] Lin F. Chen X. M. (2007). Genetics and molecular mapping of genes for race-specific all-stage resistance and non-race-specific higher-temperature adult-plant resistance to stripe rust in spring wheat cultivar Alpowa. Theor. Appl. Genet. 114, 1277–1287. 10.1007/s00122-007-0518-0 17318493

[B54] Line R. F. (2002). Stripe rust of wheat and barley in North America: a retrospective historical review. Annu. Rev. Phytopathol. 40, 75–118. 10.1146/annurev.phyto.40.020102.111645 12147755

[B55] Liu F. Gao X. Wang J. Gao C. Li X. Li X.. (2016). Transcriptome sequencing to identify transcription factor regulatory network and alternative splicing in endothelial cells under VEGF stimulation. J. Mol. Neurosci. 58, 170–177. 10.1007/s12031-015-0653-z 26395122

[B56] Liu W. Frick M. Huel R. Nykiforuk C. L. Wang X. Gaudet D. A.. (2014). The stripe rust resistance gene *Yr10* encodes an evolutionary-conserved and unique CC-NBS-LRR sequence in wheat. Mol. Plant 7, 1740–1755. 10.1093/mp/ssu112 25336565

[B57] Liu X. Zhu Y. Zhai H. Cai H. Ji W. Luo X.. (2012). AtPP2CG1, a protein phosphatase 2C, positively regulates salt tolerance of *Arabidopsis* in abscisic acid-dependent manner. Biochem. Biophys. Res. Commun. 422, 710–715. 10.1016/j.bbrc.2012.05.064 22627139

[B58] Liu Y. Zhou J. White K. P. (2014). RNA-Seq differential expression studies: more sequence or more replication? Bioinformatics 30, 301–304. 10.1093/bioinformatics/btt688 24319002 PMC3904521

[B59] Livak K. J. Schmittgen T. D. (2001). Analysis of relative gene expression data using real-time quantitative PCR and the 2(-Delta Delta C(T)) method. Methods 25, 402–408. 10.1006/meth.2001.1262 11846609

[B60] Lu H. P. (1996). High temperature resistance of xiaoyan 6 wheat to strip rust. J. Northwest Sci-Tech Univ. Agric. for (Nat. Sci. Ed.) 24, 102–104.

[B61] Lu S. Y. Li G. X. (1958). The effect of light and temperature to the strip rust of wheat cultivars. Acta Phytophy Sin. 4, 129–135.

[B62] Lu Y. Wang M. Chen X. See D. Chao S. Jing J. (2014). Mapping of *Yr62* and a small-effect QTL for high-temperature adult-plant resistance to stripe rust in spring wheat PI 192252. Theor. Appl. Genet. 127, 1449–1459. 10.1007/s00122-014-2312-0 24781075

[B63] Ma Q. Shang H. S. (2000). High-temperature resistance of wheat cultivar Xiaoyan series to wheat stripe rust. Acta Agric. Boreali Occident Sin. 9, 39–42. 10.3969/j.issn.1004-1389.2000.01.010

[B64] Maimbo M. Ohnishi K. Hikichi Y. Yoshioka H. Kiba A. (2007). Induction of a small heat shock protein and its functional roles in *Nicotiana* plants in the defense response against *Ralstonia solanacearum*. Plant Physiol. 145, 1588–1599. 10.1104/pp.107.105353 17965181 PMC2151688

[B65] Martin G. B. Bogdanove A. J. Sessa G. (2003). Understanding the functions of plant disease resistance proteins. Annu. Rev. Plant Biol. 54, 23–61. 10.1146/annurev.arplant.54.031902.135035 14502984

[B66] McCarthy D. J. Chen Y. Smyth G. K. (2012). Differential expression analysis of multifactor RNA-Seq experiments with respect to biological variation. Nucleic Acids Res. 40, 4288–4297. 10.1093/nar/gks042 22287627 PMC3378882

[B67] Milus E. A. Line R. F. (1986). Gene action for inheritance of durable, high-temperature, adult-plant resistance to stripe rust in wheat. Phytopathology 76, 435–444. 10.1094/Phyto-76-435

[B68] Moore J. W. Herrera-Foessel S. Lan C. Schnippenkoetter W. Ayliffe M. Huerta-Espino J.. (2015). A recently evolved hexose transporter variant confers resistance to multiple pathogens in wheat. Nat. Genet. 47, 1494–1498. 10.1038/ng.3439 26551671

[B69] Oono Y. Kobayashi F. Kawahara Y. Yazawa T. Handa H. Itoh T.. (2013). Characterisation of the wheat (*Triticum aestivum* L.) transcriptome by de novo assembly for the discovery of phosphate starvation-responsive genes: gene expression in Pi-stressed wheat. BMC Genomics 14:77. 10.1186/1471-2164-14-77 23379779 PMC3598684

[B70] Panstruga R. Parker J. E. Schulze-Lefert P. (2009). SnapShot: plant immune response pathways. Cell 136:978. 10.1016/j.cell.2009.02.020 19269372

[B71] Paolacci A. R. Tanzarella O. A. Porceddu E. Ciaffi M. (2009). Identification and validation of reference genes for quantitative RT-PCR normalization in wheat. BMC Mol. Biol. 10:11. 10.1186/1471-2199-10-11 19232096 PMC2667184

[B72] Peng Y. Bartley L. E. Chen X. Dardick C. Chern M. Ruan R.. (2008). OsWRKY62 is a negative regulator of basal and *Xa21*-mediated defense against *Xanthomonas oryzae* pv. oryzae in rice. Mol. Plant 1, 446–458. 10.1093/mp/ssn024 19825552

[B73] Qayoum A. Line R. F. (1985). High-temperature, adult-plant resistance to stripe rust of wheat. Phytopathology 75, 1121–1125. 10.1094/Phyto-75-1121

[B74] Ramakers C. Ruijter J. M. Deprez R. H. L. Moorman A. F. M. (2003). Assumption-free analysis of quantitative real-time polymerase chain reaction (PCR) data. Neurosci. Lett. 339, 62–66. 10.1016/S0304-3940(02)01423-4 12618301

[B75] Ren R. S. Wang M. N. Chen X. M. Zhang Z. J. (2012). Characterization and molecular mapping of *Yr52* for high-temperature adult-plant resistance to stripe rust in spring wheat germplasm PI 183527. Theor. Appl. Genet. 125, 847–857. 10.1007/s00122-012-1877-8 22562146

[B76] Risk J. M. Selter L. L. Chauhan H. Krattinger S. G. Kumlehn J. Hensel G.. (2013). The wheat *Lr34* gene provides resistance against multiple fungal pathogens in barley. Plant Biotechnol. J. 11, 847–854. 10.1111/pbi.12077 23711079

[B77] Sanseverino W. Hermoso A. D'Alessandro R. Vlasova A. Andolfo G. Frusciante L.. (2013). PRGdb 2.0: towards a community-based database model for the analysis of *R*-genes in plants. Nucleic Acids Res. 41, 1167–1171. 10.1093/nar/gks1183 23161682 PMC3531111

[B78] Scholtz J. J. Visser B. (2013). Reference gene selection for qPCR gene expression analysis of rust-infected wheat. Physiol. Mol. Plant Pathol. 81, 22–25. 10.1016/j.pmpp.2012.10.006

[B79] Schweighofer A. Hirt H. Meskiene I. (2004). Plant PP2C phosphatases: emerging functions in stress signaling. Trends Plant Sci. 9, 236–243. 10.1016/j.tplants.2004.03.007 15130549

[B80] Shang H. S. (1998). High temperature resistance of wheat to stripe rust. Sci. Agric. Sin. 31, 46–50.

[B81] Shang H. S. Wang L. G. (1997). Characteristics of expression of high-temperature resistance to strip rust in wheat. J. Plant Prot. 24, 97–100.

[B82] Shannon P. Markiel A. Ozier O. Baliga N. S. Wang J. T. Ramage D.. (2003). Cytoscape: a software environment for integrated models of biomolecular interaction networks. Genome Res. 13, 2498–2504. 10.1101/gr.1239303 14597658 PMC403769

[B83] Shimono M. Koga H. Akagi A. Hayashi N. Goto S. Sawada M.. (2012). Rice WRKY45 plays important roles in fungal and bacterial disease resistance. Mol. Plant Pathol. 13, 83–94. 10.1111/j.1364-3703.2011.00732.x 21726399 PMC6638719

[B84] Sui X. X. Wang M. N. Chen X. M. (2009). Molecular mapping of a stripe rust resistance gene in spring wheat cultivar Zak. Phytopathology 99, 1209–1215. 10.1094/PHYTO-99-10-1209 19740035

[B85] Takahashi A. Casais C. Ichimura K. Shirasu K. (2003). HSP90 interacts with RAR1 and SGT1 and is essential for *RPS2*-mediated disease resistance in *Arabidopsis*. Proc. Natl. Acad. Sci. U.S.A. 100, 11777–11782. 10.1073/pnas.2033934100 14504384 PMC208834

[B86] Tamas L. Mistrik I. Huttova J. Haluskova L. Valentovicova K. Zelinova V. (2010). Role of reactive oxygen species-generating enzymes and hydrogen peroxide during cadmium, mercury and osmotic stresses in barley root tip. Planta 231, 221–231. 10.1007/s00425-009-1042-z 19898864

[B87] Tatineni S. Wosula E. N. Bartels M. Hein G. L. Graybosch R. A. (2016). Temperature-dependent *Wsm1* and *Wsm2* gene-specific blockage of viral long-distance transport provides resistance to wheat streak mosaic virus and *Triticum mosaic virus* in wheat. Mol. Plant Microbe Interact. 29, 724–738. 10.1094/MPMI-06-16-0110-R 27551888

[B88] Trapnell C. Roberts A. Goff L. Pertea G. Kim D. Kelley D. R.. (2012). Differential gene and transcript expression analysis of RNA-Seq experiments with TopHat and Cufflinks. Nat. Protoc. 7, 562–578. 10.1038/nprot.2012.016 22383036 PMC3334321

[B89] Uauy C. Brevis J. C. Chen X. Khan I. Jackson L. Chicaiza O.. (2005). High-temperature adult-plant (HTAP) stripe rust resistance gene *Yr36* from Triticum turgidum ssp. dicoccoides is closely linked to the grain protein content locus Gpc-B1. Theor. Appl. Genet. 112, 97–105. 10.1007/s00122-005-0109-x 16208504

[B90] Wan A. M. (2003). Reviews of occurrence of wheat stripe rust disease in 2002 in China. Plant Prot. 29, 5–8. 10.3969/j.issn.0529-1542.2003.02.001

[B91] Wang B. T. Shang H. S. (2003). Histopathology and ultrastructure of wheat in expression of high-temperature resistance to stripe rust. J. Plant Prot. 30, 119–124. 10.3321/j.issn:0577-7518.2003.02.002

[B92] Wang C. F. Yin G. H. Xia X. C. He Z. H. Zhang P. P. Yao Z. J. (2016). Molecular mapping of a new temperature-sensitive gene *LrZH22* for leaf rust resistance in Chinese wheat cultivar Zhoumai 22. Mol. Breed. 36:18 10.1007/s11032-016-0437-3

[B93] Wang G. F. Wei X. Fan R. Zhou H. Wang X. Yu C.. (2011). Molecular analysis of common wheat genes encoding three types of cytosolic heat shock protein 90 (Hsp90): functional involvement of cytosolic Hsp90s in the control of wheat seedling growth and disease resistance. New Phytol. 191, 418–431. 10.1111/j.1469-8137.2011.03715.x 21488877

[B94] Wang J. J. Tao F. An F. Zou Y. P. Tian W. Chen X. M.. (2017). Wheat transcription factor TaWRKY70 is positively involved in high-temperature seedling plant resistance to *Puccinia striiformi*s f. sp. tritici. Mol. Plant Pathol. 18, 649–661. 10.1111/mpp.12425 27145738 PMC6638234

[B95] Wang J. Lan P. Gao H. Zheng L. Li W. Schmidt W. (2013). Expression changes of ribosomal proteins in phosphate- and iron-deficient *Arabidopsis* roots predict stress-specific alterations in ribosome composition. BMC Genomics 14:783. 10.1186/1471-2164-14-783 24225185 PMC3830539

[B96] Wang M. N. Chen X. M. (2017). Chapter 5: stripe rust resistance, in Stripe Rust, eds Chen X. M. Kang Z. S. (Dordrecht, ZS: Springer), 353–558.

[B97] Wang Y. Bao Z. Zhu Y. Hua J. (2009). Analysis of temperature modulation of plant defense against biotrophic microbes. Mol. Plant Microbe Interact. 22, 498–506. 10.1094/MPMI-22-5-0498 19348568

[B98] Wellings C. R. (2011). Global status of stripe rust: a review of historical and current threats. Euphytica 179, 129–141. 10.1007/s10681-011-0360-y

[B99] Xing D. H. Lai Z. B. Zheng Z. Y. Vinod K. M. Fan B. F. Chen Z. X. (2008). Stress- and pathogen-induced *Arabidopsis* WRKY48 is a transcriptional activator that represses plant basal defense. Mol. Plant 1, 459–470. 10.1093/mp/ssn020 19825553

[B100] Yao Q. Y. Xu Z. B. Wang M. N. Jing J. X. Shang H. S. (2006). Inheritance analysis of stripe rust resistance of wheat cultivar Xiaoyan 6 under the high temperature. Acta Phytophy Sin. 33, 117–121. 10.13802/j.cnki.zwbhxb.2006.02.002

[B101] Yu D. Chen C. Chen Z. (2001). Evidence for an important role of WRKY DNA binding proteins in the regulation of *NPR1* gene expression. Plant Cell 13, 1527–1540. 10.1105/tpc.13.7.1527 11449049 PMC139550

[B102] Zhang H. Yang Y. Wang C. Liu M. Li H. Fu Y.. (2014). Large-scale transcriptome comparison reveals distinct gene activations in wheat responding to stripe rust and powdery mildew. BMC Genomics 15:898. 10.1186/1471-2164-15-898 25318379 PMC4201691

[B103] Zhang Z. J. Yang G. H. Li G. H. Jin S. L. Yang X. B. (2001). Transgressive segregation, heritability, and number of genes controlling durable resistance to stripe rust in one chinese and two italian wheat cultivars. Phytopathology 91, 680–686. 10.1094/PHYTO.2001.91.7.680 18942998

[B104] Zheng W. Huang L. Huang J. Wang X. Chen X. Zhao J.. (2013). High genome heterozygosity and endemic genetic recombination in the wheat stripe rust fungus. Nat. Commun. 4:2673. 10.1038/ncomms3673 24150273 PMC3826619

[B105] Zhou X. L. Wang M. N. Chen X. M. Lu Y. Kang Z. S. Jing J. X. (2014). Identification of *Yr59* conferring high-temperature adult-plant resistance to stripe rust in wheat germplasm PI 178759. Theor. Appl. Genet. 127, 935–945. 10.1007/s00122-014-2269-z 24487945

[B106] Zhu Y. Qian W. Hua J. (2010). Temperature modulates plant defense responses through NB-LRR proteins. PLoS Pathog. 6:e1000844. 10.1371/journal.ppat.1000844 20368979 PMC2848567

