# Supplementary figures and images for: Transcriptomic Analysis Reveal the Molecular Mechanisms of Wheat Higher-Temperature Seedling-Plant Resistance to Puccinia striiformis f. sp. tritici
Source: Front Plant Sci. 2018 Feb 28;9:240. doi: 10.3389/fpls.2018.00240 (PMC5835723; doi:10.3389/fpls.2018.00240)

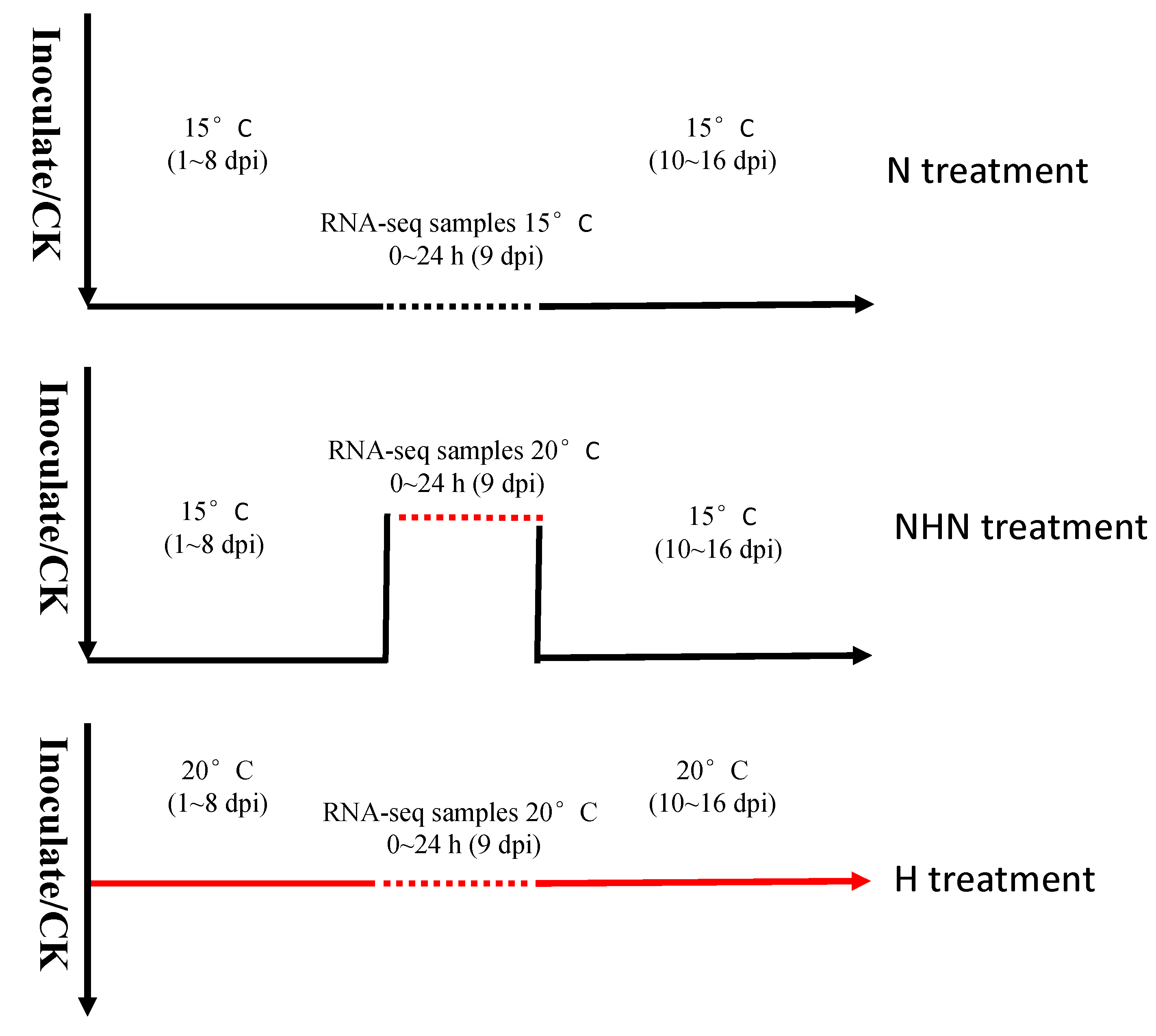

Supplement: Figure S1 — The experiment processes of three different temperature treatments. The red lines indicate the higher temperature (20°C) treatment. The black lines indicate the normal temperature (15°C) treatment. The 8~9 days post-inoculation (dpi) are equivalent to 192~216 h after inoculation. The dotted lines indicate the samples at 0 and 24 h (9 dpi) of XY 6 for RNA-Seq. N treatment: constant normal temperature (15°C); NHN treatment: switch of normal and higher temperatures (15°C~20°C~15°C); and H: constant higher temperature (20°C). CK: sterile water-inoculated wheat plants used as control for all treatments. [file Image1.TIF]

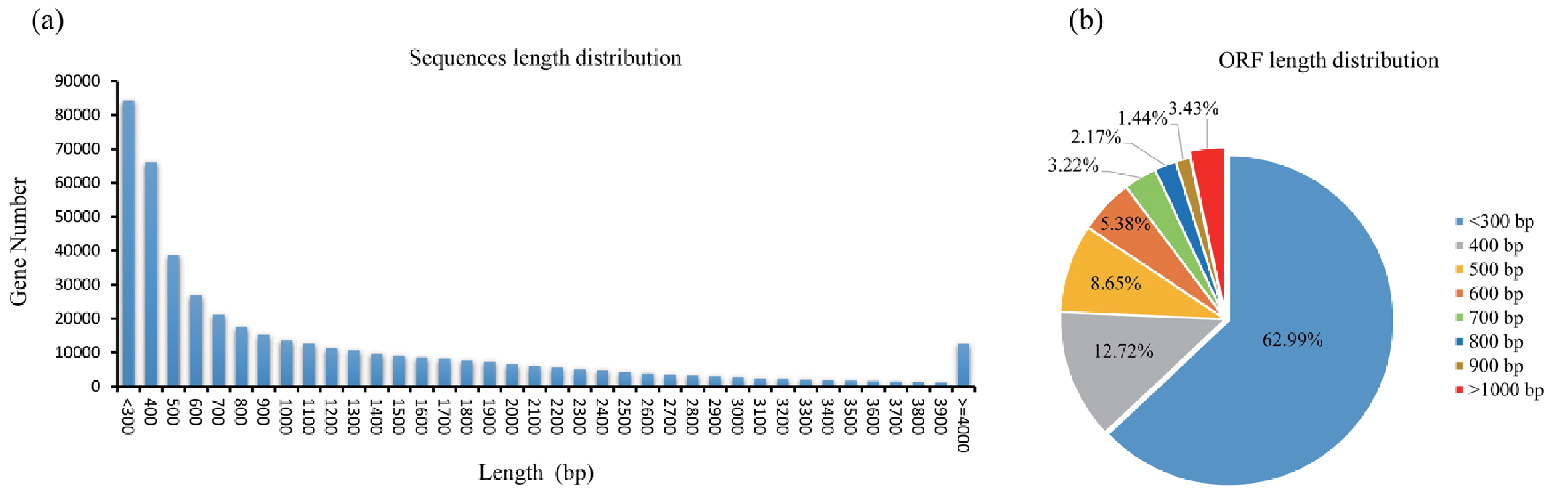

Supplement: Figure S3 — Sequences (A) and open reading form (ORF) (B) length distribution of the RNA-Seq data using the CDMC assembly. [file Image3.TIF]
