# Supplementary material for: Transcriptomic Analysis Reveal the Molecular Mechanisms of Wheat Higher-Temperature Seedling-Plant Resistance to Puccinia striiformis f. sp. tritici
Source: Front Plant Sci. 2018 Feb 28;9:240. doi: 10.3389/fpls.2018.00240 (PMC5835723; doi:10.3389/fpls.2018.00240)
Supplement: Figure S2 — Amplification efficiency and melting curve of each transcripts. ATP-dependent 26S proteasome regulatory subunit (26S, black) and cell division control (CDC, red) proteins are chosen as reference genes. The target gene is indicated in blue. [file Image2.PDF]

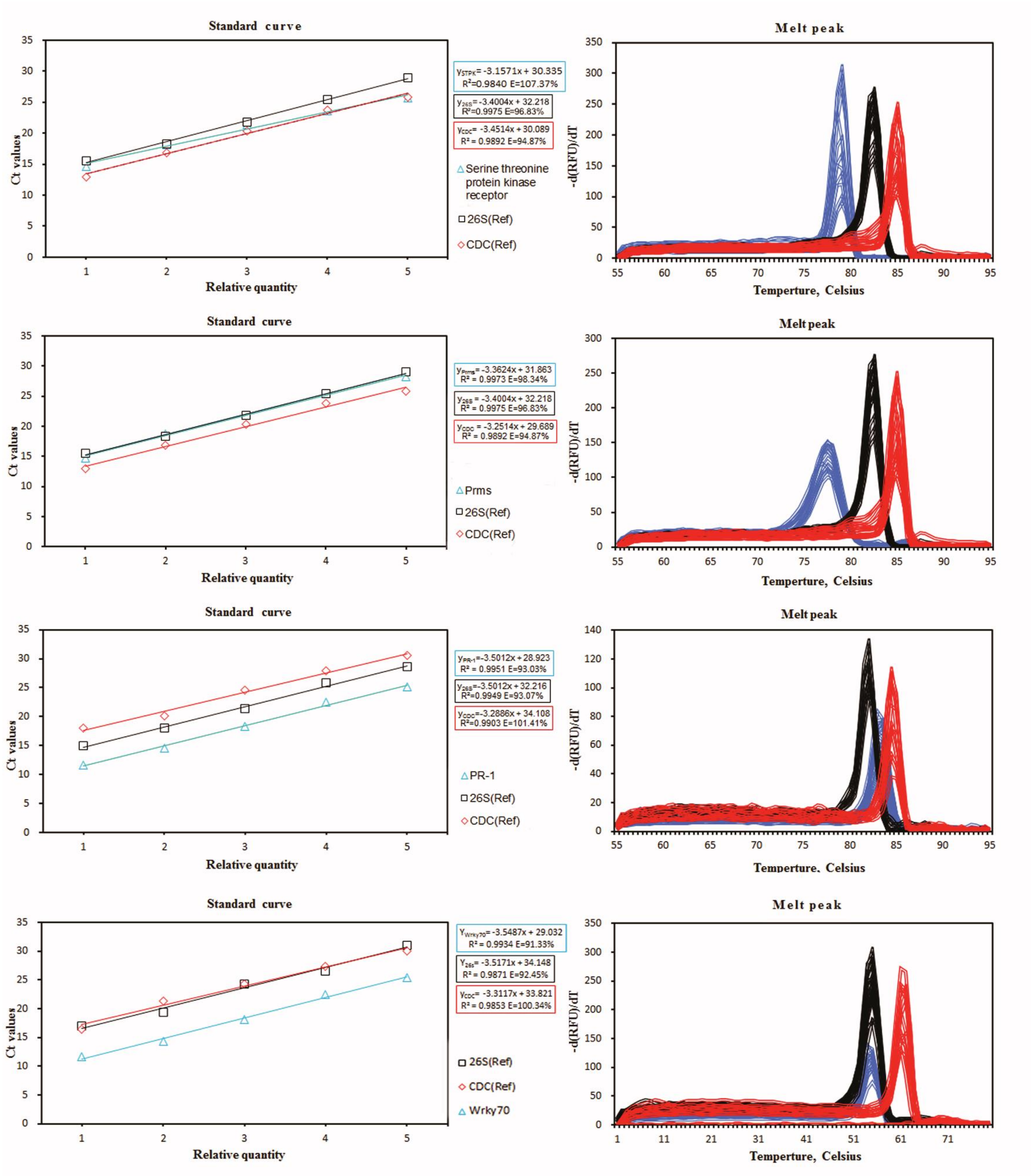
Standard curve
Ct values
Relative quantity
ySTPK= -3.1571x + 30.335 R²=0.9840 E=107.37%
y26S= -3.4004x + 32.218 R²=0.9975 E=96.83%
yCDC= -3.4514x + 30.089 R² = 0.9892 E=94.87%
Serine threonine protein kinase receptor
26S(Ref)
CDC(Ref)
Melt peak
-d(RFU)/dT
Temperture, Celsius
Standard curve
Ct values
Relative quantity
yPrms= -3.3624x + 31.863 R² = 0.9973 E=98.34%
y26S = -3.4004x + 32.218 R² = 0.9975 E=96.83%
yCDC = -3.2514x + 29.689 R² = 0.9892 E=94.87%
Prms
26S(Ref)
CDC(Ref)
Melt peak
-d(RFU)/dT
Temperture, Celsius
Standard curve
Ct values
Relative quantity
yPR-1=-3.5012x + 28.923 R² = 0.9951 E=93.03%
y26S=-3.5012x + 32.216 R²=0.9949 E=93.07%
yCDC=-3.2886x + 34.108 R²=0.9903 E=101.41%
PR-1
26S(Ref)
CDC(Ref)
Melt peak
-d(RFU)/dT
Temperture, Celsius
Standard curve
Ct values
Relative quantity
YWrky70= -3.5487x + 29.032 R² = 0.9934 E=91.33%
Y26s= -3.5171x + 34.148 R² = 0.9871 E=92.45%
yCDC= -3.3117x + 33.821 R² = 0.9853 E=100.34%
26S(Ref)
CDC(Ref)
Wrky70
Melt peak
-d(RFU)/dT
Temperture, Celsius

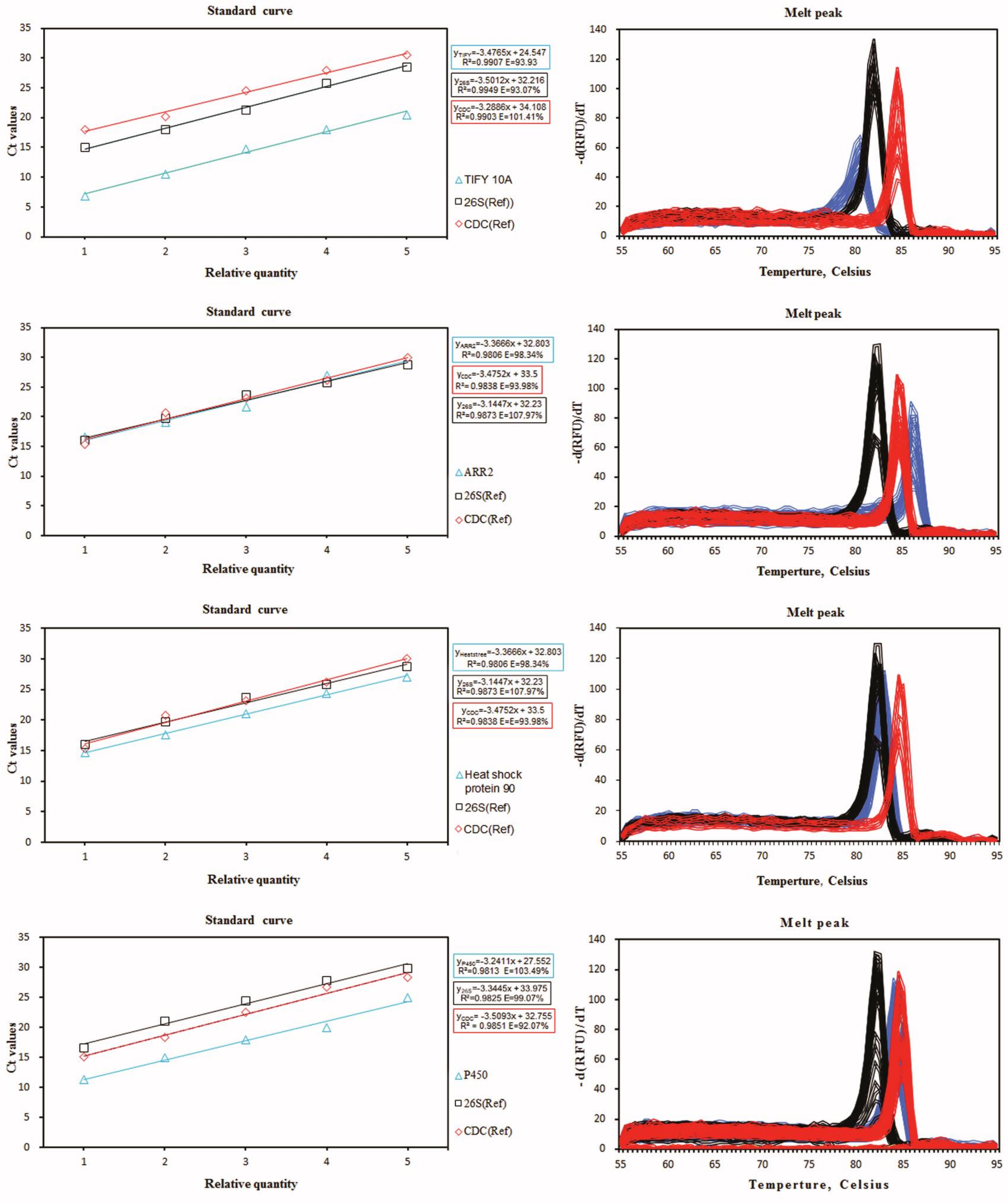
Standard curve
Ct values
0 5 10 15 20 25 30 35
1 2 3 4 5
Relative quantity
y_TIFY=-3.4765x + 24.547
R²=0.9907 E=93.93
y_26S=-3.5012x + 32.216
R²=0.9949 E=93.07%
y_CDC=-3.2886x + 34.108
R²=0.9903 E=101.41%
TIFY 10A
26S(Ref))
CDC(Ref)
Melt peak
-d(RFU)/dT
0 20 40 60 80 100 120 140
55 60 65 70 75 80 85 90 95
Temperture, Celsius
Standard curve
Ct values
0 5 10 15 20 25 30 35
1 2 3 4 5
Relative quantity
y_ARR2=-3.3666x + 32.803
R²=0.9806 E=98.34%
y_CDC=-3.4752x + 33.5
R²= 0.9838 E=93.98%
y_26S=-3.1447x + 32.23
R²=0.9873 E=107.97%
ARR2
26S(Ref)
CDC(Ref)
Melt peak
-d(RFU)/dT
0 20 40 60 80 100 120 140
55 60 65 70 75 80 85 90 95
Temperture, Celsius
Standard curve
Ct values
0 5 10 15 20 25 30 35
1 2 3 4 5
Relative quantity
y_Heatstree=-3.3666x + 32.803
R²=0.9806 E=98.34%
y_26S=-3.1447x + 32.23
R²=0.9873 E=107.97%
y_CDC=-3.4752x + 33.5
R²=0.9838 E=E=93.98%
Heat shock
protein 90
26S(Ref)
CDC(Ref)
Melt peak
-d(RFU)/dT
0 20 40 60 80 100 120 140
55 60 65 70 75 80 85 90 95
Temperture, Celsius
Standard curve
Ct values
0 5 10 15 20 25 30 35
1 2 3 4 5
Relative quantity
y_P450=-3.2411x + 27.552
R²=0.9813 E=103.49%
y_26S=-3.3445x + 33.975
R²=0.9825 E=99.07%
y_CDC= -3.5093x + 32.755
R² = 0.9851 E=92.07%
P450
26S(Ref)
CDC(Ref)
Melt peak
-d(R FU)/dT
0 20 40 60 80 100 120 140
55 60 65 70 75 80 85 90 95
Temperture, Celsius

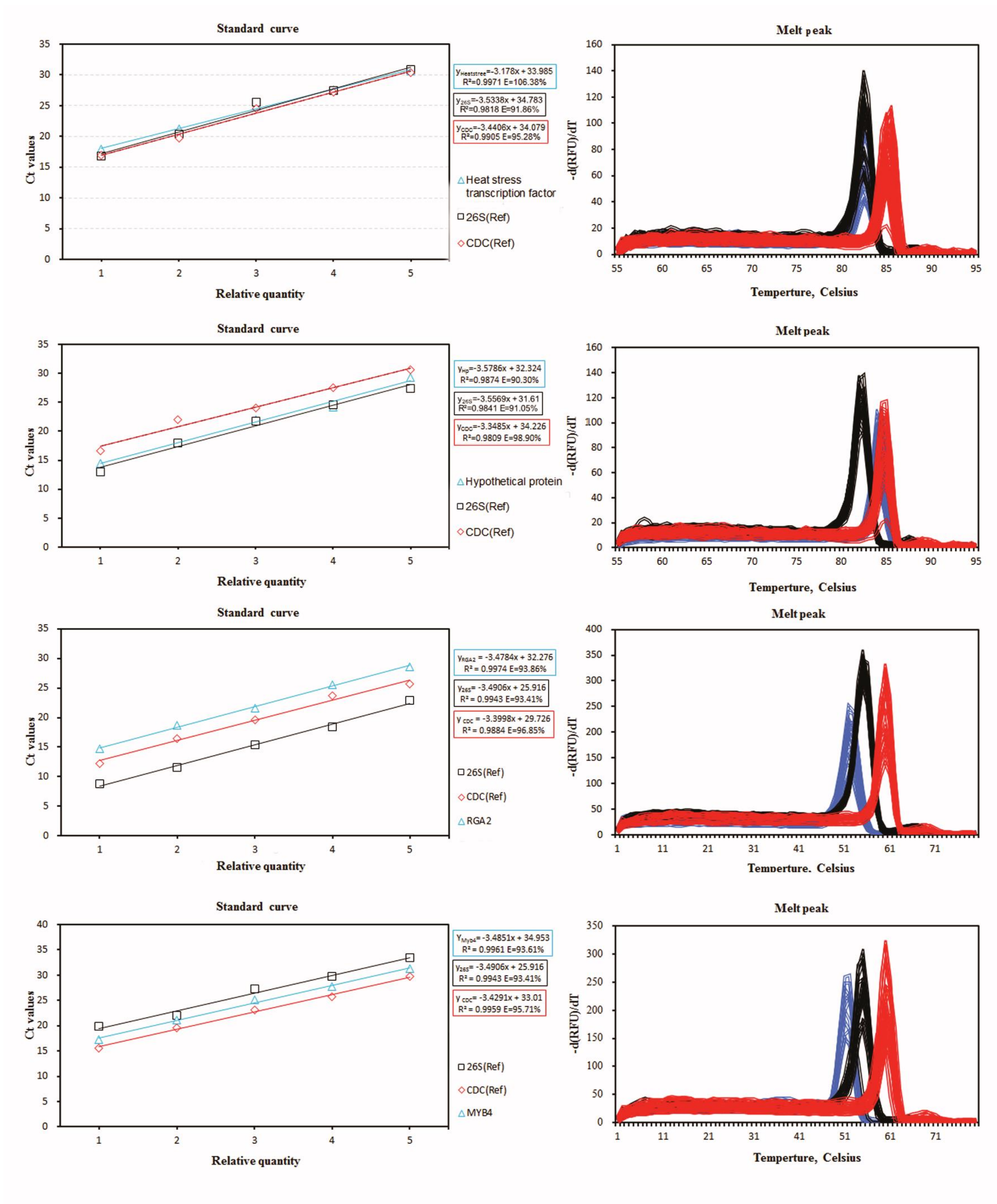

FIGURE S2 | Amplification efficiency and melting curve of each transcripts. ATP-dependent 26S proteasome regulatory subunit (26S, black) and cell division control (CDC, red) proteins are chosen as reference genes. The target gene is indicated in blue.
