# Supplementary material for: Transcriptomic Analysis Reveal the Molecular Mechanisms of Wheat Higher-Temperature Seedling-Plant Resistance to Puccinia striiformis f. sp. tritici
Source: Front Plant Sci. 2018 Feb 28;9:240. doi: 10.3389/fpls.2018.00240 (PMC5835723; doi:10.3389/fpls.2018.00240)
Supplement: Table S1 — Kolmogorov-Smirnov test for each treated sample. [file Table1.DOCX]

**TABLE S1 ׀ Kolmogorov-Smirnov test for each treated sample.**

| **Sample** | **SD** | **Sig.** | **Data distribution type** |
| --- | --- | --- | --- |
| I-N-24 | 0.310 | .000 | Normal distribution |
| I-NHN-24 | 0.340 | .000 | Normal distribution |
| I-H-24 | 0.200 | .000 | Normal distribution |
| NI-N-24 | 0.160 | .000 | Normal distribution |
| NI-NHN-24 | 0.210 | .000 | Normal distribution |
| NI-H-24 | 0.250 | .000 | Normal distribution |

**TABLE S2 ׀ List of primers used in qRT-PCR.**

| **Express transcripts** |  | **Forward primer (5'-3')** |  | **Reverse primer (5'-3')** |
| --- | --- | --- | --- | --- |
| RGA2/TR141761\|c0_g2_i2 | RGA2-F | GGAGCAGATGGTGGAGCAGAC | RGA2-R | CGAGTAGGAGGAGAGGGTGAACA |
| MYB4/ TCONS_00152941 | MYB4-F | AACGAGGCGGAATAGCAA | MYB4-R | AAGCAAAGCGAGTAGTAATAAACA |
| Heat stress transcription factor a-4d/TR108629\|c1_g1_i1 | HSTF-F | GAAGTTGTTGTGCTTGAAGT | HSTF-R | CTACGAGATGGTGGAGGA |
| Serine threonine-protein kinase receptor/TR224404\|c0_g1_i1 | STPKR-F | TATCACGTTCCGCCACCT | STPKR-R | GATTTCCCGAGCAAGAGC |
| Pathogenesis-related PR-1 proteins/TR140243\|c2_g2_i1 | PR-1-F | GGACGCCTACAACTGAACC | PR-1-R | GTTTGGAGCGGCTTTAT |
| ARR2/ TR167267\|c1_g1_i1 | ARR2-F | CTTACATAGCTCGGAGAAATACAG | ARR2-R | GCCCACAAGGCATAACAG |
| Hypothetical protein/TR95380\|c1_g1_i1 | HP-F | CGTCGTTGATGGAGATGCT | HP-R | AGGAGGACCAAGAACAAAGAA |
| P450/ TR191560\|c0_g1_i1 | P450-F | GTGGCTCCTTTCGGACTACC | P450-R | GGGCGACTTTGAGTTCTTGC |
| Heat shock protein 90/TR212876\|c0_g1_i2 | HS90-F | AGGTAGATGGGGTAGGAGATGA | HS90-R | GCACAACGATGACGAGCAGT |
| TIFY 10A/TR196955\|c1_g1_i3 | TIFY 10A-F | AGCCTGCTCTTCCTCTTCGC | TIFY 10A-R | AGGACGGGGCCTGAGAGAGC |
| Prms/TR235666\|c2_g1_i6 | Prms-F | CTCGCCGAAGATAGCAGCAC | Prms-R | CGTTGTCGTCCCAGGTCAC |
| Wrky70/TCONS_00006459 | Wrky70-F | GGGTTGTCCCACCGTGTC | Wrky70-R | CCCCGACTTTCGCTTCC |

**TABLE S3 ׀ Q30 quality levels of 30 RNA-Seq samples.**

| **No** | **Sample** | **Total reads number** | **Q30(%)** |
| --- | --- | --- | --- |
| 1 | I-H-0-1 | 25167263 | 92.81 |
| 2 | I-H-0-2 | 24452234 | 92.34 |
| 3 | I-H-0-3 | 19511903 | 90.72 |
| 4 | I-H-24-1 | 18390752 | 90.62 |
| 5 | I-H-24-2 | 27490451 | 92.7 |
| 6 | I-H-24-3 | 22859773 | 92.23 |
| 7 | I-N-0-1 | 20075674 | 88.62 |
| 8 | I-N-0-2 | 20123805 | 91.58 |
| 9 | I-N-0-3 | 26077533 | 92.79 |
| 10 | I-N-24-1 | 26081348 | 91.06 |
| 11 | I-N-24-2 | 22727327 | 92.92 |
| 12 | I-N-24-3 | 19391420 | 92.98 |
| 13 | I-NHN-24-1 | 19001128 | 91.36 |
| 14 | I-NHN-24-2 | 24078338 | 92.73 |
| 15 | I-NHN-24-3 | 25328783 | 90.49 |
| 16 | NI-H-0-1 | 25179856 | 91.19 |
| 17 | NI-H-0-2 | 19128545 | 91.77 |
| 18 | NI-H-0-3 | 23557885 | 91.25 |
| 19 | NI-H-24-1 | 24006697 | 91.61 |
| 20 | NI-H-24-2 | 18495707 | 92.07 |
| 21 | NI-H-24-3 | 22088328 | 91.37 |
| 22 | NI-N-0-1 | 19149018 | 86.14 |
| 23 | NI-N-0-2 | 25166466 | 92.07 |
| 24 | NI-N-0-3 | 16544909 | 91.6 |
| 25 | NI-N-24-1 | 19438753 | 86.33 |
| 26 | NI-N-24-2 | 23723019 | 92.25 |
| 27 | NI-N-24-3 | 17750568 | 91.47 |
| 28 | NI-NHN-24-1 | 24122689 | 92.48 |
| 29 | NI-NHN-24-2 | 19258129 | 90.33 |
| 30 | NI-NHN-24-3 | 21100390 | 86.1 |
|  |  | Sum (659468691) | Ave (91.226) |

**TABLE S4 ׀ Statistics of *de novo*, mapping and CDMC assembly strategies.**

| **Metrics** | ***De novo*** | **Mapping** | **CDMC** |
| --- | --- | --- | --- |
| Number of sequences | 588794 | 274188 | 445226 |
| Average length | 818 | 1656 | 1083 |
| N50 length | 1276 | 2280 | 1849 |

**TABLE S5 ׀ Statistical table of differentially expressed genes (DEGs) and annotated DEGs from the CDMC assembly.**

| **Type** | **NHN vs N (I*T)** | **H vs NHN (I*T)** |
| --- | --- | --- |
| Total_num DEGs | 3596 | 5379 |
| Up | 1788 | 2278 |
| Down | 1808 | 3101 |
| Nr | 2758 | 4430 |
| SwissProt | 1932 | 2862 |
| GO | 1774 | 3264 |
| KEGG | 663 | 972 |

Data are evaluated by using linear model (1). FDR < 0.05, log_2_-fold change > 1 or < -1 and logCPM (log_2_ counts per million) > -2 were used for analysis. I and T means the inoculation and temperature factors, respectively.

* means the interaction of factors.

**TABLE S6 ׀ Go characterisation of differentially expressed genes (DEGs) responding to *Puccinia striiformis* f. sp. *tritici* under the normal-higher-normal (NHN) temperature treatment.**

| **GO categorie** | **GO ID** | **GO term** | **DEGs with GO annotation** | **All genes with GO annotation** | **Corrected**  ***P*-value** |
| --- | --- | --- | --- | --- | --- |
| **Cellular Component** | GO:0031224 | Intrinsic component of membrane | 272, 54.6% | 37920, 42.1% | 0.00000123 |
|  | GO:0016021 | Integral component of membrane | 271, 54.4% | 37791, 42.0% | 0.00000142 |
|  | GO:0044425 | Membrane part | 278, 55.8% | 39593, 44.0% | 0.00000685 |
|  | GO:0016020 | Membrane | 326, 65.5% | 48495, 53.8% | 0.00000923 |
|  | GO:0005575 | Cellular_component | 304, 61.0% | 44852, 49.8% | 0.0000291 |
|  | GO:0002178 | Palmitoyl transferase complex | 3, 0.6% | 6, 0.0% | 0.00035 |
|  | GO:0017059 | Serine C-palmitoyl transferase complex | 3, 0.6% | 6, 0.0% | 0.00035 |
|  | GO:0031211 | Endoplasmic reticulum palmitoyl transferase complex | 3, 0.6% | 6, 0.0% | 0.00035 |
|  | GO:0000322 | Storage vacuole | 3, 0.6% | 9, 0.0% | 0.00147 |
| **Molecular Function** | GO:0004672 | Protein kinase activity | 130, 19.9% | 12187, 10.7% | 4.89E-10 |
|  | GO:0004674 | Protein serine/threonine kinase activity | 86, 13.2% | 6694, 5.9% | 6.29E-10 |
|  | GO:0016773 | Phosphotransferase activity, alcohol group as acceptor | 132, 20.2% | 13396, 11.7% | 6.68E-08 |
|  | GO:0016301 | Kinase activity | 147, 22.5% | 15979, 14.0% | 0.000000552 |
|  | GO:0030246 | Carbohydrate binding | 39, 6.0% | 2480, 2.2% | 0.00000531 |
|  | GO:0005315 | Inorganic phosphate transmembrane transporter activity | 6, 0.9% | 63, 0.1% | 0.00041 |
|  | GO:0004758 | Serine C-palmitoyl transferase activity | 3, 0.5% | 6, 0.0% | 0.00087 |
|  | GO:0016454 | C-palmitoyl transferase activity | 3, 0.5% | 6, 0.0% | 0.00087 |
|  | GO:0016772 | Transferase activity, transferring phosphorus-containing groups | 151, 23.2% | 18798, 16.4% | 0.00138 |
|  | GO:0003735 | Structural constituent of ribosome | 32, 4.9% | 2479, 2.2% | 0.00543 |
|  | GO:0005524 | ATP binding | 164, 25.2% | 21311, 18.6% | 0.00546 |
|  | GO:0032559 | Adenyl ribonucleotide binding | 188, 28.8% | 25120, 22.0% | 0.0057 |
|  | GO:0030554 | Adenyl nucleotide binding | 188, 28.8% | 25134, 22.0% | 0.0059 |
|  | GO:0015114 | Phosphate ion transmembrane transporter activity | 4, 0.6% | 31, 0.0% | 0.007 |
|  | GO:0043167 | Ion binding | 328, 50.3% | 48527, 42.5% | 0.00742 |
|  | GO:0046509 | 1,2-diacylglycerol 3-beta-galactosyltransferase activity | 3, 0.5% | 15, 0.0% | 0.01917 |
|  | GO:0016408 | C-acyl transferase activity | 3, 0.5% | 18, 0.0% | 0.03394 |
|  | GO:0005198 | Structural molecule activity | 33, 5.1% | 2871, 2.5% | 0.03627 |
|  | GO:0001883 | Purine nucleoside binding | 193, 29.6% | 26786, 23.4% | 0.03943 |
|  | GO:0032550 | Purine ribonucleoside binding | 193, 29.6% | 26786, 23.4% | 0.03943 |
|  | GO:0032555 | Purine ribonucleotide binding | 193, 29.6% | 26786, 23.4% | 0.03943 |
|  | GO:0017076 | Purine nucleotide binding | 193, 29.6% | 26812, 23.5% | 0.04162 |
|  | GO:0035639 | Purine ribonucleoside triphosphate binding | 169, 25.9% | 22977, 20.1% | 0.04403 |
|  | GO:0009978 | Allene oxide synthase activity | 2, 0.3% | 4, 0.0% | 0.04643 |
|  | GO:0032549 | Ribonucleoside binding | 193, 29.6% | 26898, 23.5% | 0.04971 |
| **Biological Process** | GO:0006468 | Protein phosphorylation | 128, 22.3% | 11994, 11.0% | 4.76E-12 |
|  | GO:0009069 | Serine family amino acid metabolic process | 89, 15.5% | 7866, 7.2% | 7.55E-09 |
|  | GO:0016310 | Phosphorylation | 152, 26.5% | 17476, 16.0% | 9.32E-08 |
|  | GO:0019538 | Protein metabolic process | 230, 40.1% | 30710, 28.2% | 0.000000416 |
|  | GO:0006817 | Phosphate ion transport | 10, 1.7% | 125, 0.1% | 0.00000127 |
|  | GO:0044267 | Cellular protein metabolic process | 189, 32.9% | 24906, 22.8% | 0.0000163 |
|  | GO:1901605 | Alpha-amino acid metabolic process | 102, 17.8% | 11311, 10.4% | 0.0000439 |
|  | GO:0006464 | Cellular protein modification process | 145, 25.3% | 19005, 17.4% | 0.00117 |
|  | GO:0036211 | Protein modification process | 145, 25.3% | 19005, 17.4% | 0.00117 |
|  | GO:0006796 | Phosphate-containing compound metabolic process | 170, 29.6% | 23285, 21.4% | 0.0016 |
|  | GO:0008037 | Cell recognition | 16, 2.8% | 728, 0.7% | 0.00188 |
|  | GO:0048544 | Recognition of pollen | 16, 2.8% | 728, 0.7% | 0.00188 |
|  | GO:0009875 | Pollen-pistil interaction | 16, 2.8% | 739, 0.7% | 0.00227 |
|  | GO:0006793 | Phosphorus metabolic process | 170, 29.6% | 23494, 21.5% | 0.00279 |
|  | GO:0006520 | Cellular amino acid metabolic process | 104, 18.1% | 12706, 11.7% | 0.00297 |
|  | GO:1901564 | Organonitrogen compound metabolic process | 168, 29.3% | 23342, 21.4% | 0.00455 |
|  | GO:0007166 | Cell surface receptor signaling pathway | 16, 2.8% | 880, 0.8% | 0.01947 |
|  | GO:0031407 | Oxylipin metabolic process | 7, 1.2% | 174, 0.2% | 0.03501 |
|  | GO:0009856 | Pollination | 16, 2.8% | 951, 0.9% | 0.04853 |
|  | GO:0044706 | Multi-multicellular organism process | 16, 2.8% | 951, 0.9% | 0.04853 |
